# Supplementary material for: Prevalence of Trichomonas vaginalis infection and protozoan load in South African women: a cross-sectional study
Source: BMJ Open. 2017 Oct 8;7(10):e016959. doi: 10.1136/bmjopen-2017-016959 (PMC5640031; doi:10.1136/bmjopen-2017-016959)
Supplement: Author's manuscript [file bmjopen-2017-016959.draft_revisions.pdf]

BMJ Open

BMJ Open is committed to open peer review. As part of this commitment we make the peer review history of every article we publish publicly available.

When an article is published we post the peer reviewers' comments and the authors' responses online. We also post the versions of the paper that were used during peer review. These are the versions that the peer review comments apply to.

The versions of the paper that follow are the versions that were submitted during the peer review process. They are not the versions of record or the final published versions. They should not be cited or distributed as the published version of this manuscript.

BMJ Open is an open access journal and the full, final, typeset and author-corrected version of record of the manuscript is available on our site with no access controls, subscription charges or pay-per-view fees (http://bmjopen.bmj.com).

If you have any questions on BMJ Open's open peer review process please email editorial.bmjopen@bmj.com

BMJ Open

Prevalence of Trichomonas vaginalis infection and protozoan load in South African women

Journal:	*BMJ Open*
Manuscript ID	bmjopen-2017-016959
Article Type:	Research
Date Submitted by the Author:	31-Mar-2017
Complete List of Authors:	de Waaij, Dewi; VU University Medical Centre, Laboratory of Immunogenetics, Dept. Medical Microbiology and Infection Control<br>Dubbink, Jan Henk; Anova Health Institute; University of Maastricht, Institute for Public Health Genomics, Faculty of Health, Medicine and LIfe Sciences<br>Ouburg, Sander; VU University Medical Centre, Laboratory of Immunogenetics, Dept. Medical Microbiology and Infection Control<br>Peters, Remco; Anova Health Institute, ; Maastricht University Medical Centre, Medical Microbiology<br>Morre, S; VU University medical center, Pathology, Laboratory of Immunogenetics
**Primary Subject Heading**:	Infectious diseases
Secondary Subject Heading:	Epidemiology, Infectious diseases, Sexual health
Keywords:	EPIDEMIOLOGY, INFECTIOUS DISEASES, Molecular diagnostics < INFECTIOUS DISEASES

SCHOLARONE™
Manuscripts

For peer review only

Prevalence of *Trichomonas vaginalis* infection and protozoan load in South African women

Dewi J. de Waaij[1,2] , Jan Henk Dubbink[1,2], Sander Ouburg[1*], Remco P.H. Peters[3,4], Servaas A. Morré[1,2]

1 VU University Medical Centre, Department of Medical Microbiology & Infection Control, Laboratory of Immunogenetics, Amsterdam, The Netherlands.

2 Institute for Public Health Genomics (IPHG), Department of Genetics and Cell Biology, Research School GROW (School for Oncology & Developmental Biology), Faculty of Health, Medicine & Life Sciences, University of Maastricht, Maastricht, The Netherlands.

3 Anova Health Institute, Johannesburg and Tzaneen, South Africa.

4 Department of Medical Microbiology, University of Maastricht, Maastricht, The Netherlands.

* Corresponding author

dr. S. Ouburg, VU University Medical Center, Laboratory of Immunogenetics, dept. Medical Microbiology and Infection Control, De Boelelaan 1108, 1081HZ Amsterdam, the Netherlands. Tel.: +31 (0)20 444 8417, Fax.: +31 (0)20 444 8418. E-mail: s.ouburg@vumc.nl

Keywords: **Trichomonas vaginalis, risk factors, protozoan load, microbiological characteristics, rural South Africa**

Word count: 2584

Journal: Sexual Transmitted Infections

For peer review only

ABSTRACT

Objectives. *Trichomonas vaginalis* is thought to be the most common non-viral sexually transmitted infection (STI) worldwide. We investigated the prevalence, risk factors and protozoan load of *Trichomonas vaginalis* (*T. vaginalis* load) infection in South African women.

Methods. A cross-sectional study of 604 women was conducted at 25 primary healthcare facilities in rural South Africa (Mopani district). *T. vaginalis* DNA was detected in vaginal and rectal swabs. In a uni- and multivariate analyses the *T. vaginalis* infection was investigated in relation to demographic characteristics, medical history, and behavioural factors. The *T. vaginalis* load was determined as the logarithm of DNA copies per microliter sample solution.

Results. Collected vaginal and rectal swabs were tested for *T. vaginalis* DNA. Prevalence of vaginal *T. vaginalis* was 20% (95% CI 17.0-23.4) and rectal 1.2% (95% CI 0.6-2.4). Most women (66%) with a vaginal infection were asymptomatic. Factors associated with *T. vaginalis* infection were a relationship status of single (OR, 2.4; 95% CI, 1.5-4.0; *P* < 0.001) and HIV positive-infection (OR, 1.6; 95% CI, 1.0-2.6; *P* = 0.041). Women with vaginal *T. vaginalis* infection were more likely to have concurrent *C. trachomatis* rectal infection than those without vaginal infection (12% vs. 3%; p<0.001, OR: 4.1). A higher median *T. vaginalis* load was observed among women with observed vaginal discharge compared to those without vaginal discharge (*P* = 0.025).

Conclusions. Vaginal trichomoniasis is highly prevalent in rural South Africa, especially among single women and those with HIV infection, and often presents without symptoms.

Key messages

- Vaginal *T. vaginalis* is highly prevalent in South Africa
- Women with observed vaginal discharge were more likely to have a higher *T. vaginalis* load compared to women without discharge
- Women with vaginal *T. vaginalis* infection were more likely to have concurrent *C. trachomatis* rectal infection than those without vaginal infection

Data sharing: All data of this study have been described in the paper. No additional data for this study are available.

For peer review only

Author contributions:

DJdW: Laboratory analyses, statistical analyses, writing of the manuscript

JHD: Sample collection, writing of the manuscript

SO: Statistical analyses, reviewing of the manuscript

RPHP: Sample collection, reviewing of the manscript, study supervision

SAM: reviewing of the manuscript, study supervision.

For peer review only

INTRODUCTION

Trichomonas vaginalis is a protozoan parasite that causes trichomoniasis and is mostly sexually transmitted. It is the most common non-viral sexually transmitted infection (STI) worldwide (1). The infection is asymptomatic in 85% of the women and in 77% of the men(1). The WHO describes that *T. vaginalis* in the African region occurs ten times as often in women than in men(2). Vaginal *T. vaginalis* infections in the entire African region is estimated to be 42.8 million(2). If the infection is symptomatic, women may report a change in vaginal discharge, intermenstrual bleeding, or vaginal blood loss during or after sexual contact. Other symptoms that can occur are vaginal itching, dysuria, and abdominal pain. Also upper reproductive tract disease syndromes can occur, including pelvic inflammatory disease. Women with a *T. vaginalis* infection have 4.7-fold increase in the risk of PID(3-5) and tubal pathology(6). Women infected with *T. vaginalis* also have a 1.3-fold increase in the risk of preterm labour(5).

In women, *T. vaginalis* infection can persist for months and, if left untreated, it can increase the risk for HIV acquisition if exposed(6;7). *T. vaginalis* could lead to an increase in the vaginal HIV load and thus potentially increase the risk of HIV transmission to a sexual partner(8). In addition, an association between *T. vaginalis* and *Chlamydia trachomatis* infection has been found(9). Having a concurrent chlamydial infection was a predictor of both prevalent and incident *T. vaginalis*.

Despite the estimated large burden of *T. vaginalis* infection, data on clinical presentation, demographic and behavioural factors associated with infection, and microbiological factors is relatively limited. *T. vaginalis* prevalence has been reported for a few African countries and ranges from 6.5-40%(10). One of the explanations for the often high *T. vaginalis* prevalence is the lack of STI screening programmes and limited control measurements. The estimated burden of disease is significant but available data of symptomatology, co-infections and pathogen load are limited. *C. trachomatis* and *Neisseria gonorrhoeae* are other important causative micro-organisms for STI, showing an association with the pathogen load as compared to the clinical presentation and the development of long-term sequelae(11;12).

In this study, we present the epidemiology of *T. vaginalis* infection including the *T. vaginalis* load, as measured by a validated nucleic acid amplification technique. We studied a high HIV prevalence setting in rural South Africa.

For peer review only

METHODS

Population and Design

This study was part of a cross-sectional study of 604 women in rural Mopani District, South Africa, as described previously(13). In brief, women aged 18-49 years old who reported sexual activity during the prior 6 months were recruited at 25 primary healthcare (PHC) facilities, regardless of the reason for visiting the facility that day. After informed consent, demographic characteristics, medical history and details of sexual behaviour were collected using a questionnaire. Healthcare worker collected vaginal and rectal swabs were obtained for molecular testing of *T. vaginalis*.

The Human Ethics Research Committee of the University of the Witwatersrand, South Africa, approved the study (Ref M110726).

Laboratory testing

Collected vaginal and rectal swabs were analysed at the laboratory of Medical Microbiology & Infection Control, VU University Medical Centre, Amsterdam, the Netherlands. High Pure PCR Template Preparation Kit (HPPTP) (Roche Diagnostics, Basel, Switzerland) was used for the extraction of bacterial DNA. Detection of *T. vaginalis* DNA was done with the Presto^{plus} assay (Microbiome Ltd., Amsterdam, the Netherlands) and the LightCycler II 480 (Roche Diagnostics) was used for the quantitative PCR for *C. trachomatis*, *N. gonorrhoeae* and *T. vaginalis*(14). PCR positivity was determined using the Cp (crossing point) value: we used a threshold Cp of <38 for a sample to be classified as positive. Samples with Cp values of ≥38 were retested and if again ≥38 were excluded from further analyses (indeterminate samples).

Data analyses

The following risk factors were used in our analyses: age, being single, unemployment, using hormonal contraceptives, current pregnancy, history of vaginal discharge syndrome (VDS), currently being pregnant, the use of hormonal contraceptives, administering intravaginal cleansing, visiting bars, alcohol use, concurrent partners, condom use during last sex act, partner >10 years older, experiencing coercion and/or force, and sex for money or for other benefits(13). Coercion reflects sex inequality in relationships which is associated with increased risk(15).

Data were double-entered in Epi Data (Epi Info^{TM} version 3.5.3) by two researchers independently, were compared for inconsistencies, cleaned and verified. The data were analysed using SPSS version 20.0 (SPSS Inc., Chicago, USA). Dichotomous data were compared using χ^2 test or Fisher's Exact test and continuous data using the Mann-Whitney *U* test. Odds ratios (ORs) with 95% confidence interval

For peer review only - http://bmjopen.bmj.com/site/about/guidelines.xhtml

(CI) were calculated. Vaginal *T. vaginalis* infection was defined symptomatic when either abnormal vaginal discharge during physical examination, intermenstrual bleeding, or vaginal blood loss during or after sexual intercourse was reported. Univariate analysis was performed to examine possible associations of demographic variables and clinical and behavioural factors in relation to *T. vaginalis*. Multivariate analysis was conducted using a backward selection procedure and likelihood ratio tests. As a proxy for the *T. vaginalis* load we used the median Cp value, which was calculated for each risk factor and compared between infected versus non-infected women.

For peer review only

RESULTS

Demographics of study population

A quarter of all women (n= 154; 25.5%) had an age ≤24, over half of the women were single (334; (55.3%), 26 women reported anal intercourse (4.3%), a high proportion was unemployed, and almost 30% reported to be infected with HIV (177; 29.3%). For more details see **Table 1**.

Prevalence of *T. vaginalis* infection

Of the 604 vaginal samples, one sample was excluded based on lack of biological material and 28 gave an indeterminate result (2 times Cp values of ≥38); these were excluded resulting in 575 samples for analysis. For vaginal infection*,* 113/575 (20%) samples tested positive.
Of the 604 rectal samples, nine samples gave an indeterminate result (2 times Cp values of ≥38) and were excluded resulting in 595 samples. For rectal infection*,* 7/595 (1.2%) samples tested positive. Four patients were infected *T. vaginalis* at both the rectal and vaginal site.

Clinical manifestation of infection

Symptoms of vaginal infection were reported by 196 (34%) of the women. Of these, 38 had *T. vaginalis* infection meaning 38/113 (34%) of the women with TV reported symptoms. Of the 462 women without *T. vaginalis,* 158 reported symptoms of infection (34%). None of the individual symptoms (vaginal discharge, intermenstrual bleeding and vaginal blood loss related to sexual contact) was associated with *T. vaginalis* infection. For rectal infection (n=7) the numbers were low and no rectal symptomatology was scored.

Factors associated with *Trichomonas vaginalis* infection

In univariate analyses we observed significant associations for single status, being HIV positive, the use of hormonal contraceptives, and visiting bars (**Table 1**). In multivariate analysis (**Table 1**), having a relationship status that is single was significantly associated with a vaginal *T. vaginalis* infection (adjusted OR [aOR], 2.4 [95% CI, 1.5-4.0, p <0.001]) and presence of HIV infection was significantly associated (aOR, 1.6; [95% CI 1.0-2.6; p = 0.041]) *T. vaginalis* infection. Within women with a current vaginal *T. vaginalis* infection, a rectal *C. trachomatis* infection is significantly more present (P<0.001, OR: 4.1).

For peer review only

Table 1. Univariate and Multivariate Analysis of Factors associated with Vaginal *Trichomonas vaginalis* Infection in South African Women

			Univariate		Multivariate	
Factor	n	%	OR (95% CI)	*P*	OR (95% CI)	*P*
Age ≤24	154	25.5	0.9 (0.6-1.6)	0.95	-	NS
Single	334	55.3	2.4 (1.5-3.9)	**<0.001**	2.4 (1.5-4.0)	**<0.001**
Unemployed	424	70.2	1.5 (0.9-2.4)	0.135	-	NS
HIV infected	177	29.3	1.6 (1.0-2.4)	**0.040**	1.6 (1.0-2.6)	**0.041**
History of VDS*	196	32.4	1.0 (0.7-1.6)	0.878	-	NS
Currently pregnant	94	15.9	1.6 (1.0-2.7)	0.067	-	NS
Hormonal contraceptives	230	33.6	0.6 (0.4-0.9)	**0.016**	-	NS
Intravaginal cleansing	132	21.9	1.0 (0.6-1.6)	0.841	-	NS
Visits bars	76	12.6	1.8 (1.0-3.1)	**0.027**	1.7 (0.96-3.1)	NS
Alcohol use	12	2	1.4 (0.4-5.1)	0.640	-	NS
Concurrent partners	85	14	1.1 (0.7-1.6)	0.799	-	NS
Condom use last act	207	34.3	1.2 (0.8-1.8)	0.487	-	NS
Partner >10 y older	202	33.4	1.2 (0.8-1.9)	0.335	-	NS
Experienced coercion**	169	28	0.7 (0.4-1.1)	0.142	0.6 (0.4-1.0)	0.058
Experienced force	38	6.3	1.3 (0.6-2.8)	0.541	-	NS
Sex for money or other benefits	10	1.7	1.3 (0.5-3.3)	0.579	-	NS

**VDS contains reported change in vaginal discharge, intermenstrual bleeding, or vaginal blood loss during or after sexual contact*
***Coercion reflects sex inequality in relationships, associated with increased risk*(13)

For peer review only

Determinants of *T. vaginalis* load

The median Cp value for vaginal *T. vaginalis* load of infection was 28.0 (range 17.0 – 37.9). We did not observe any significant associations between the *T. vaginalis* load and demographic factors (**Table 2**). Also no association was found between the *T. vaginalis* load and symptoms in general. However, in the group of women with *T. vaginalis* infection who had vaginal discharge observed during examination, a significantly higher load was observed as compared to those without vaginal discharge (*P* = 0.025).

Table 2. *T. vaginalis* load in association with risk factors

Risk factor		Median Cp	*P*
Age ≤24	≤24	28	0.747
	>24	27.7	
Single	Yes	27.3	0.971
	No	28	
Unemployed	Yes	27.7	0.810
	No	29.7	
HIV infected	Yes	27.5	0.986
	No	27.8	
History of VDS	Yes	28	0.954
	No	27.6	
Currently pregnant	Yes	28	0.794
	No	27.7	
Hormonal contraceptives	Yes	27.6	0.464
	No	28	
Intravaginal cleansing	Yes	28.5	0.433
	No	27.7	
Visits bars	Yes	25.3	0.310
	No	28	
Alcohol use	Yes	27.9	0.613
	No	30.2	
Concurrent partners	Yes	27	0.335
	No	28	
Condom use last sex act	Yes	27	0.339
	No	28	
Experiences force	Yes	29	0.603
	No	27.9	

For peer review only

DISCUSSION

In this study, we describe the prevalence, risk factors, and the load of *T. vaginalis* infection. This is one of a few studies to report these aspects for *T. vaginalis* in a cohort from South Africa. We observed a vaginal *T. vaginalis* prevalence of 20% (95% CI 17.0-23.4) and a rectal prevalence of 1.2% (95% CI 0.6-2.4).

In multivariate analysis two risk factors for vaginal *T. vaginalis* infection were identified: having a relationship status that is single and the presence of HIV infection. In addition, within women with vaginal *T. vaginalis* infection, a rectal *C. trachomatis* infection is significantly more present. Finally, a high *T. vaginalis* load appears to be associated with vaginal discharge observed during examination.

The prevalence of vaginal *T. vaginalis* infection in our study corresponds with the range reported by other studies in Africa (3.2%-40%)(10;16). Rectal *T. vaginalis* infections are rarely described. One study from San Francisco, USA, describes a rectal *T. vaginalis* prevalence of 0.6%, based on 'men who have sex with men' (MSM) who reported receptive anal sex within six months prior to their clinic visit(17).

We show two demographic factors that may contribute to the high vaginal *T. vaginalis* prevalence in our study. First, we observed that a *T. vaginalis* infection is associated with a single relationship status. Joffe *et al.* described that a single marital status is often associated with having more than one sexual partner and thereby an increased risk for STI in general(18). To be more specific, it has been demonstrated that women having more than two concurrent male sexual partners are more likely to have a *T. vaginalis* infection(19). Although we were not able to confirm this, we hypothesize that a single marital status is associated with having more than one concurrent sexual partner.

It has been suggested that *T. vaginalis* infection are age-tied. Women who were older than 20 years of age, were more likely to be infected with *T. vaginalis*(20). In our study we did not observe an association between age an T. vaginalis infections.

We confirm an association of positive HIV status with presence of *T. vaginalis* infection, which has been reported by several other studies(4;8;21;22). *T. vaginalis* infection was present in almost a quarter of HIV-infected women in our study. This is in line with the previously described higher trichomonas prevalence in high HIV prevalence cities compared to low HIV prevalence cities(10;16). The biological link between HIV and TV is yet unclear, however bacterial vaginosis, is described to be an independent risk factor for acquisition of STI, and acquisition and transmission of HIV(23). Bacterial vaginosis is described to be associated with a 1.5-2-fold increased risk for incident trichomonal infection(24). This could suggest an association between HIV and *T. vaginalis* where an asymptomatic *T. vaginalis* infection could increase the risk of HIV infection.

For peer review only

It has been described that non-sexual transmission of *T. vaginalis* occurs in girls who did not have sexual intercourse(25). A striking prevalence found by a study among women from Ndola, Zambia, who denied ever had sexual intercourse was 43%(10). In those cases, *T. vaginalis* would be transmitted by wet clothes and water, since some studies suggest that *T. vaginalis* can survive up to days in warm mineral water and also for hours in urine(25). *T. vaginalis* is described to have some kind of robustness, which may explain its different transmission ways, compared to other STI. This may be an explanation of many hypotheses for the high prevalence, however, they need to be further explored.

Twenty-six percent of all women reported symptoms, without being diagnosed with a *T. vaginalis* infection. Although 20% of these women were diagnosed with another vaginal STI than *T. vaginalis*, symptoms are evidently a poor predictor for a *T. vaginalis* infection. We did not find a significant difference in symptomatology and *T. vaginalis* infection status. More knowledge about *T. vaginalis* disease would provide valuable information for potential targeted treatment approaches versus syndromic management.

In general, self-reported symptoms are not reliable in predicting an STI, like *T. vaginalis*(26;27). However, 31 (16%) of the women vaginally infected with *T. vaginalis* were co-infected with either *C. trachomatis* or *N. gonorrhoeae* (data not shown). Symptoms of these frequent occurring STI may show comparable symptoms and should be taken into consideration. In this study we did not observe an increased risk for coinfection with *C. trachomatis* and/or *N. gonorrhoeae* in *T. vaginalis* infected patients (data not shown). However, in a previous study from our group we describe an increased risk of obtaining a *T. vaginalis* coinfection when infected with either *C. trachomatis* or *N. gonorrhoeae*.

This is one of a few studies to report aspects of rectal *T. vaginalis* in a cohort from South Africa. We observed that rectal *C. trachomatis* infections are significant more present in combination with a vaginal *T. vaginalis* infection. This may be due to several factors. First, sexual behaviour including anal sex is an important factor that may play a role in the presence of co-infections. Second, there is the ability to get re-infected via the gastrointestinal tract by auto inoculation, after being cured of the vaginal infection for Chlamydia, but it is unclear if this route of infection is also present for *T. vaginalis* infections and thus if the rectal site could be the reservoir for *T. vaginalis* infection(28). These potential transmission routes may result from vaginal wiping techniques, poor personal hygiene, using fingers during sex or for cleansing afterward, and use of sex toys(13). The most likely explanation is that the rectal samples may be contaminated by cervicovaginal fluid due to the close anatomical proximity(29;30) and that *T. vaginalis* positive rectal samples in women were contaminated with *T. vaginalis* positive vaginal fluid of the perianal surface(31).

For peer review only

It is conceivable that microorganism load may affect the clinical course of infection. It has been shown in studies of another STI, *C. trachomatis*, that there is an association between the bacterial load and genital manifestations of infection(32;33). A higher *C. trachomatis* bacterial load is associated with the presence of at least two specific symptoms (vaginal discharge, irregular bleeding, or pelvic or abdominal pain). The impact of *T. vaginalis* load has not yet been described. We hypothesized that *T. vaginalis* load may affect the clinical course of the infection and compared several factors with the load of *T. vaginalis*. We did not find any significant association between the load and reported symptoms of a *T. vaginalis* infection. However, within women with observed vaginal discharge during examination, a significantly higher load was found. The load determination was performed using Cp values of the *T. vaginalis* PCR. Another technique to determine the average load per human is to incorporate the number of human cells. Our results prove that there is an association between *T. vaginalis* load and discharge. However, the literature on *T. vaginalis* load is scarce, and the effect of the load has to be further explored.

Although this is a unique study about the epidemiology and microbiological characteristics of *T. vaginalis* in rural South Africa with a relatively good sample size, a limitation of our study is that the number of rectal T. vaginalis infections is rather small for further analyses. As a consequence, this could have played a role in our statistical analyses. In addition, there can be a selection bias based on for instance the geographical inclusion, and self-reported symptoms can be more subjective than clinician based symptomatology.

In conclusion, *T. vaginalis* infections are highly prevalent in the rural South African population investigated. The asymptomatic nature, potential for non-sexual transmission especially in socio-economic poor settings, and the syndromic management approach to STI control in this region are potential factors contributing to the prevalence. We observed a low rectal prevalence of which might be contamination with *T. vaginalis* positive vaginal fluid of the perianal surface. In multivariate analyses being single or HIV positive were identified as risk factors for vaginal *T. vaginalis* infection. Finally, a high *T. vaginalis* load seems associated with vaginal discharge observed during examination.

For peer review only

Funding

This work was supported by the Dutch Society for Tropical Medicine (NVTG), the Netherlands. The Anova Health Institute is supported by the US President's Emergency Plan for AIDS Relief program via the US Agency for International Development under Cooperative Agreement No. AID-674-A-12-00015. The views expressed in this manuscript do not necessarily reflect those of the President's Emergency Plan for AIDS Relief or the US Agency for International Development.

CONFLICT OF INTEREST

S.A.M. is a fulltime employee of the VU University Medical Center Amsterdam, and has been involved in the technical development of the Presto^{plus} CT-NG-TV assay (Microbiome, The Netherlands) via Microbiome Ltd. (S.A.M. is co-founder and co-director), a spin-off company of the VU University Medical Center, Amsterdam, the Netherlands.

None of the other authors report a potential conflict of interest.

For peer review only

References

(1) Kissinger P. Epidemiology and treatment of trichomoniasis. Curr Infect Dis Rep 2015 June;17(6):484.

(2) World Health Organization, Department of Reproductive Health and Research. Global incidence and prevalence of selected curable sexually transmitted infections - 2008. 2012.

(3) Moodley P, Wilkinson D, Connolly C, Moodley J, Sturm AW. Trichomonas vaginalis is associated with pelvic inflammatory disease in women infected with human immunodeficiency virus. Clin Infect Dis 2002 February 15;34(4):519-22.

(4) Kissinger P, Adamski A. Trichomoniasis and HIV interactions: a review. Sex Transm Infect 2013 September;89(6):426-33.

(5) Cotch MF, Pastorek JG, Nugent RP, Hillier SL, Gibbs RS, Martin DH et al. Trichomonas vaginalis associated with low birth weight and preterm delivery. The Vaginal Infections and Prematurity Study Group. Sex Transm Dis 1997 July;24(6):353-60.

(6) Soper D. Trichomoniasis: under control or undercontrolled? Am J Obstet Gynecol 2004 January;190(1):281-90.

(7) Van Der Pol B, Williams JA, Orr DP, Batteiger BE, Fortenberry JD. Prevalence, incidence, natural history, and response to treatment of Trichomonas vaginalis infection among adolescent women. J Infect Dis 2005 December 15;192(12):2039-44.

(8) Van Der Pol B. Trichomonas vaginalis infection: the most prevalent nonviral sexually transmitted infection receives the least public health attention. Clin Infect Dis 2007 January 1;44(1):23-5.

(9) Helms DJ, Mosure DJ, Metcalf CA, Douglas JM, Jr., Malotte CK, Paul SM et al. Risk factors for prevalent and incident Trichomonas vaginalis among women attending three sexually transmitted disease clinics. Sex Transm Dis 2008 May;35(5):484-8.

(10) Buve A, Weiss HA, Laga M, Van DE, Musonda R, Zekeng L et al. The epidemiology of trichomoniasis in women in four African cities. AIDS 2001 August;15 Suppl 4:S89-S96.

(11) Michel CE, Sonnex C, Carne CA, White JA, Magbanua JP, Nadala EC, Jr. et al. Chlamydia trachomatis load at matched anatomic sites: implications for screening strategies. J Clin Microbiol 2007 May;45(5):1395-402.

(12) Bissessor M, Tabrizi SN, Fairley CK, Danielewski J, Whitton B, Bird S et al. Differing Neisseria gonorrhoeae bacterial loads in the pharynx and rectum in men who have sex with men: implications for gonococcal detection, transmission, and control. J Clin Microbiol 2011 December;49(12):4304-6.

(13) Peters RP, Dubbink JH, van der Eem L, Verweij SP, Bos ML, Ouburg S et al. Cross-sectional study of genital, rectal, and pharyngeal Chlamydia and gonorrhea in women in rural South Africa. Sex Transm Dis 2014 September;41(9):564-9.

For peer review only

(14) de Waaij DJ, Ouburg S, Dubbink JH, Peters RP, Morre SA. Evaluation of Presto(plus) assay and LightMix kit Trichomonas vaginalis assay for detection of Trichomonas vaginalis in dry vaginal swabs. J Microbiol Methods 2016 August;127:102-4.

(15) Hoffman S, O'Sullivan LF, Harrison A, Dolezal C, Monroe-Wise A. HIV risk behaviors and the context of sexual coercion in young adults' sexual interactions: results from a diary study in rural South Africa. Sex Transm Dis 2006 January;33(1):52-8.

(16) Naidoo S, Wand H. Prevalence and incidence of Trichomonas vaginalis infections in women participating in a clinical trial in Durban, South Africa. Sex Transm Infect 2013 September;89(6):519-22.

(17) Dubbink JH, de Waaij DJ, Bos M, van der Eem L, Bebear C, Mbambazela N et al. MICROBIOLOGICAL CHARACTERISTICS OF CHLAMYDIA TRACHOMATIS AND NEISSERIA GONORRHOEAE INFECTIONS IN SOUTH AFRICAN WOMEN. J Clin Microbiol 2015 October 28.

(18) Joffe GP, Foxman B, Schmidt AJ, Farris KB, Carter RJ, Neumann S et al. Multiple partners and partner choice as risk factors for sexually transmitted disease among female college students. Sex Transm Dis 1992 September;19(5):272-8.

(19) Miller M, Liao Y, Gomez AM, Gaydos CA, D'Mellow D. Factors associated with the prevalence and incidence of Trichomonas vaginalis infection among African American women in New York city who use drugs. J Infect Dis 2008 February 15;197(4):503-9.

(20) Kwena Z, Sharma A, Wamae N, Muga C, Bukusi E. Provider characteristics among staff providing care to sexually transmitted infection self-medicating patients in retail pharmacies in Kibera slum, Nairobi, Kenya. Sex Transm Dis 2008 May;35(5):480-3.

(21) Sorvillo F, Smith L, Kerndt P, Ash L. Trichomonas vaginalis, HIV, and African-Americans. Emerg Infect Dis 2001 November;7(6):927-32.

(22) Chun HM, Carpenter RJ, Macalino GE, Crum-Cianflone NF. The Role of Sexually Transmitted Infections in HIV-1 Progression: A Comprehensive Review of the Literature. J Sex Transm Dis 2013;2013:176459.

(23) Brotman RM. Vaginal microbiome and sexually transmitted infections: an epidemiologic perspective. J Clin Invest 2011 December;121(12):4610-7.

(24) Brotman RM, Klebanoff MA, Nansel TR, Yu KF, Andrews WW, Zhang J et al. Bacterial vaginosis assessed by gram stain and diminished colonization resistance to incident gonococcal, chlamydial, and trichomonal genital infection. J Infect Dis 2010 December 15;202(12):1907-15.

(25) Neinstein LS, Goldenring J, Carpenter S. Nonsexual transmission of sexually transmitted diseases: an infrequent occurrence. Pediatrics 1984 July;74(1):67-76.

(26) Nagarajan K, Godbole S, Ramakrishnan L, Mainkar M, Ramesh S, Ganju D et al. Self-Report of STI Symptoms, Inconsistent Condom use and Condom Nonuse are

For peer review only

Poor Predictors of STI Prevalence among Men who have Sex with Men. Journal of AIDS & Clinical Research 2013 January 6.

(27) van der Eem L, Dubbink JH, Struthers HE, McIntyre JA, Ouburg S, Morre SA et al. Evaluation of syndromic management guidelines for treatment of sexually transmitted infections in South African women. Trop Med Int Health 2016 September;21(9):1138-46.

(28) Rank RG, Yeruva L. Hidden in plain sight: chlamydial gastrointestinal infection and its relevance to persistence in human genital infection. Infect Immun 2014 April;82(4):1362-71.

(29) Francis SC, Kent CK, Klausner JD, Rauch L, Kohn R, Hardick A et al. Prevalence of rectal Trichomonas vaginalis and Mycoplasma genitalium in male patients at the San Francisco STD clinic, 2005-2006. Sex Transm Dis 2008 September;35(9):797-800.

(30) Crucitti T, Jespers V, Mulenga C, Khondowe S, Vandepitte J, Buve A. Trichomonas vaginalis is highly prevalent in adolescent girls, pregnant women, and commercial sex workers in Ndola, Zambia. Sex Transm Dis 2010 April;37(4):223-7.

(31) Cosentino LA, Campbell T, Jett A, Macio I, Zamborsky T, Cranston RD et al. Use of nucleic acid amplification testing for diagnosis of anorectal sexually transmitted infections. J Clin Microbiol 2012 June;50(6):2005-8.

(32) Jalal H, Verlander NQ, Kumar N, Bentley N, Carne C, Sonnex C. Genital chlamydial infection: association between clinical features, organism genotype and load. J Med Microbiol 2011 July;60(Pt 7):881-8.

(33) Gomes JP, Borrego MJ, Atik B, Santo I, Azevedo J, Brito de SA et al. Correlating Chlamydia trachomatis infectious load with urogenital ecological success and disease pathogenesis. Microbes Infect 2006 January;8(1):16-26.

For peer review only

BMJ Open

Prevalence of Trichomonas vaginalis infection and protozoan load in South African women

Journal:	*BMJ Open*
Manuscript ID	bmjopen-2017-016959.R1
Article Type:	Research
Date Submitted by the Author:	15-Jun-2017
Complete List of Authors:	de Waaij, Dewi; VU University Medical Centre, Laboratory of Immunogenetics, Dept. Medical Microbiology and Infection Control<br>Dubbink, Jan Henk; Anova Health Institute; University of Maastricht, Institute for Public Health Genomics, Faculty of Health, Medicine and LIfe Sciences<br>Ouburg, Sander; VU University Medical Centre, Laboratory of Immunogenetics, Dept. Medical Microbiology and Infection Control<br>Peters, Remco; Anova Health Institute, ; Maastricht University Medical Centre, Medical Microbiology<br>Morre, S; VU University medical center, Pathology, Laboratory of Immunogenetics
**Primary Subject Heading**:	Infectious diseases
Secondary Subject Heading:	Epidemiology, Infectious diseases, Sexual health
Keywords:	EPIDEMIOLOGY, INFECTIOUS DISEASES, Molecular diagnostics < INFECTIOUS DISEASES

SCHOLARONE™
Manuscripts

Prevalence of *Trichomonas vaginalis* infection and protozoan load in South African women

Dewi J. de Waaij[1,2] , Jan Henk Dubbink[1,2], Sander Ouburg[1*], Remco P.H. Peters[3,4], Servaas A. Morré[1,2]

1 VU University Medical Centre, Department of Medical Microbiology & Infection Control, Laboratory of Immunogenetics, Amsterdam, The Netherlands.

2 Institute for Public Health Genomics (IPHG), Department of Genetics and Cell Biology, Research School GROW (School for Oncology & Developmental Biology), Faculty of Health, Medicine & Life Sciences, University of Maastricht, Maastricht, The Netherlands.

3 Anova Health Institute, Johannesburg and Tzaneen, South Africa.

4 Department of Medical Microbiology, University of Maastricht, Maastricht, The Netherlands.

* Corresponding author

dr. S. Ouburg, VU University Medical Center, Laboratory of Immunogenetics, dept. Medical Microbiology and Infection Control, De Boelelaan 1108, 1081HZ Amsterdam, the Netherlands. Tel.: +31 (0)20 444 8417, Fax.: +31 (0)20 444 8418. E-mail: s.ouburg@vumc.nl

Keywords: **Trichomonas vaginalis, risk factors, protozoan load, microbiological characteristics, rural South Africa**

Word count: 2584

Journal: Sexual Transmitted Infections

For peer review only

ABSTRACT

Objectives. *Trichomonas vaginalis* is thought to be the most common non-viral sexually transmitted infection (STI) worldwide. We investigated the prevalence, risk factors and protozoan load of *Trichomonas vaginalis* (*T. vaginalis* load) infection in South African women.

Methods. A cross-sectional study of 604 women was conducted at 25 primary healthcare facilities in rural South Africa (Mopani district). *T. vaginalis* DNA was detected in vaginal and rectal swabs. In a uni- and multivariate analyses the *T. vaginalis* infection was investigated in relation to demographic characteristics, medical history, and behavioural factors. The *T. vaginalis* load was determined as the logarithm of DNA copies per microliter sample solution.

Results. Collected vaginal and rectal swabs were tested for *T. vaginalis* DNA. Prevalence of vaginal *T. vaginalis* was 20% (95% CI 17.0-23.4) and rectal 1.2% (95% CI 0.6-2.4). Most women (66%) with a vaginal infection were asymptomatic. Factors associated with *T. vaginalis* infection were a relationship status of single (OR, 2.4; 95% CI, 1.5-4.0; $P < 0.001$) and HIV positive-infection (OR, 1.6; 95% CI, 1.0-2.6; $P = 0.041$). Women with vaginal *T. vaginalis* infection were more likely to have concurrent *C. trachomatis* rectal infection than those without vaginal infection (12% vs. 3%; $p<0.001$, OR: 4.1). A higher median *T. vaginalis* load was observed among women with observed vaginal discharge compared to those without vaginal discharge ($P = 0.025$).

Conclusions. Vaginal trichomoniasis is highly prevalent in rural South Africa, especially among single women and those with HIV infection, and often presents without symptoms.

Key messages

- Vaginal *T. vaginalis* is highly prevalent in South Africa
- Women with observed vaginal discharge were more likely to have a higher *T. vaginalis* load compared to women without discharge
- Women with vaginal *T. vaginalis* infection were more likely to have concurrent *C. trachomatis* rectal infection than those without vaginal infection

Data sharing: All data of this study have been described in the paper. No additional data for this study are available.

For peer review only

Author contributions:

DJdW: Laboratory analyses, statistical analyses, writing of the manuscript

JHD: Sample collection, writing of the manuscript

SO: Statistical analyses, reviewing of the manuscript

RPHP: Sample collection, reviewing of the manuscript, study supervision

SAM: reviewing of the manuscript, study supervision.

For peer review only

INTRODUCTION

Trichomonas vaginalis is a protozoan parasite that causes trichomoniasis and is mostly sexually transmitted. It is the most common non-viral sexually transmitted infection (STI) worldwide (1). The infection is asymptomatic in 85% of the women and in 77% of the men(1). The WHO describes that *T. vaginalis* in the African region occurs ten times as often in women than in men(2). Vaginal *T. vaginalis* infections in the entire African region is estimated to be 42.8 million(2). If the infection is symptomatic, women may report a change in vaginal discharge, intermenstrual bleeding, or vaginal blood loss during or after sexual contact. Other symptoms that can occur are vaginal itching, dysuria, and abdominal pain. Also upper reproductive tract disease syndromes can occur, including pelvic inflammatory disease. Women with a *T. vaginalis* infection have 4.7-fold increase in the risk of PID(3-5) and tubal pathology(6). Women infected with *T. vaginalis* also have a 1.3-fold increase in the risk of preterm labour(5).

In women, *T. vaginalis* infection can persist for months and, if left untreated, it can increase the risk for HIV acquisition if exposed(6, 7). *T. vaginalis* could lead to an increase in the vaginal HIV load and thus potentially increase the risk of HIV transmission to a sexual partner(8). In addition, an association between *T. vaginalis* and *Chlamydia trachomatis* infection has been found(9). Having a concurrent chlamydial infection was a predictor of both prevalent and incident *T. vaginalis*.

Despite the estimated large burden of *T. vaginalis* infection in the African region, data on clinical presentation, demographic and behavioural factors associated with infection, and microbiological factors is relatively limited. However, in other continents, multiple studies have been done in the context of *T. vaginalis* infections(10-13). *T. vaginalis* prevalence has been reported for a few African countries and ranges from 6.5-40%(14). One of the explanations for the often high *T. vaginalis* prevalence is the lack of STI screening programmes and limited control measurements. The estimated burden of disease is significant but available data of symptomatology, co-infections and pathogen load are limited. *C. trachomatis* and *Neisseria gonorrhoeae* are other important causative micro-organisms for STI, showing an association with the pathogen load as compared to the clinical presentation and the development of long-term sequelae(15, 16).

In this study, we present the epidemiology of *T. vaginalis* infection including the *T. vaginalis* load, as measured by a validated nucleic acid amplification technique. We studied a high HIV prevalence setting in rural South Africa.

For peer review only

METHODS

Population and Design

This study was part of a cross-sectional study of 604 women in rural Mopani District, South Africa, as described previously(17). In brief, women aged 18-49 years old who reported sexual activity during the prior 6 months were recruited at 25 primary healthcare (PHC) facilities, regardless of the reason for visiting the facility that day. After informed consent, demographic characteristics, medical history and details of sexual behaviour were collected using a questionnaire. Healthcare worker collected vaginal and rectal swabs were obtained for molecular testing of *T. vaginalis*.

The Human Ethics Research Committee of the University of the Witwatersrand, South Africa, approved the study (Ref M110726).

Laboratory testing

Collected vaginal and rectal swabs were analysed at the laboratory of Medical Microbiology & Infection Control, VU University Medical Centre, Amsterdam, the Netherlands. High Pure PCR Template Preparation Kit (HPPTP) (Roche Diagnostics, Basel, Switzerland) was used for the extraction of bacterial DNA. Detection of *T. vaginalis* DNA was done with the Presto^{plus} assay (Microbiome Ltd., Amsterdam, the Netherlands) and the LightCycler II 480 (Roche Diagnostics) was used for the quantitative PCR for *C. trachomatis*, *N. gonorrhoeae* and *T. vaginalis*(18). PCR positivity was determined using the Cp (crossing point) value: we used a threshold Cp of <38 for a sample to be classified as positive. Samples with Cp values of ≥38 were retested and if again ≥38 were excluded from further analyses (indeterminate samples).

Data analyses

The following risk factors were used in our analyses: age, being single, unemployment, current pregnancy, history of vaginal discharge syndrome (VDS), currently being pregnant, the use of hormonal contraceptives, administering intravaginal cleansing, visiting bars, alcohol use, concurrent partners, condom use during last sex act, partner >10 years older, experiencing coercion and/or force, and sex for money or for other benefits(17). Coercion reflects sex inequality in relationships which is associated with increased risk(19).

Data were double-entered in Epi Data (Epi Info^{TM} version 3.5.3) by two researchers independently, were compared for inconsistencies, cleaned and verified. The data were analysed using SPSS version 20.0 (SPSS Inc., Chicago, USA). Dichotomous data were compared using χ^2 test or Fisher's Exact test and continuous data using the Mann-Whitney *U* test. Odds ratios (ORs) with 95% confidence interval

(CI) were calculated. Vaginal *T. vaginalis* infection was defined symptomatic when either abnormal vaginal discharge during physical examination, intermenstrual bleeding, or vaginal blood loss during or after sexual intercourse was reported. Univariate analysis was performed to examine possible associations of demographic variables and clinical and behavioural factors in relation to *T. vaginalis*. Multivariate analysis was conducted using a backward selection procedure and likelihood ratio tests. As a proxy for the *T. vaginalis* load we used the median Cp value, which was calculated for each risk factor and compared between infected versus non-infected women.

For peer review only

RESULTS

Demographics of study population

A quarter of all women (n= 154; 25.5%) had an age 18-24, over half of the women were single (334; (55.3%), 26 women reported anal intercourse (4.3%), a high proportion was unemployed, and almost 30% reported to be infected with HIV (177; 29.3%). For more details see **Table 1**.

Prevalence of *T. vaginalis* infection

Of the 604 vaginal samples, one sample was excluded based on lack of biological material and 28 gave an indeterminate result (2 times Cp values of ≥38); these were excluded resulting in 575 samples for analysis. For vaginal infection*,* 113/575 (20%) samples tested positive.
Of the 604 rectal samples, nine samples gave an indeterminate result (2 times Cp values of ≥38) and were excluded resulting in 595 samples. For rectal infection*,* 7/595 (1.2%) samples tested positive. Four patients were infected *T. vaginalis* at both the rectal and vaginal site.

Clinical manifestation of infection

Symptoms of vaginal infection were reported by 196 (34%) of the women. Of these, 38 had *T. vaginalis* infection meaning 38/113 (34%) of the women with TV reported symptoms. Of the 462 women without *T. vaginalis,* 158 reported symptoms of infection (34%). None of the individual symptoms (vaginal discharge, intermenstrual bleeding and vaginal blood loss related to sexual contact) was associated with *T. vaginalis* infection. For rectal infection (n=7) the numbers were low and no rectal symptomatology was scored.

Factors associated with *Trichomonas vaginalis* infection

In univariate analyses we observed significant associations for single status, being HIV positive, the use of hormonal contraceptives, and visiting bars (**Table 1**). In multivariate analysis (**Table 1**), having a relationship status that is single was significantly associated with a vaginal *T. vaginalis* infection (adjusted OR [aOR], 2.4 [95% CI, 1.5-4.0, p <0.001]) and presence of HIV infection was significantly associated (aOR, 1.6; [95% CI 1.0-2.6; p = 0.041]) *T. vaginalis* infection. Within women with a current vaginal *T. vaginalis* infection, a rectal *C. trachomatis* infection is significantly more present (P<0.001, OR: 4.1).

For peer review only

Table 1. Univariate and Multivariate Analysis of Factors associated with Vaginal *Trichomonas vaginalis* Infection in South African Women

			Univariate		Multivariate	
Factor	n	%	OR (95% CI)	*P*	OR (95% CI)	*P*
Age 18-24	154	25.5	0.9 (0.6-1.6)	0.95	-	NS
Single	334	55.3	2.4 (1.5-3.9)	**<0.001**	2.4 (1.5-4.0)	**<0.001**
Unemployed	424	70.2	1.5 (0.9-2.4)	0.135	-	NS
HIV infected	177	29.3	1.6 (1.0-2.4)	**0.040**	1.6 (1.0-2.6)	**0.041**
History of VDS*	196	32.4	1.0 (0.7-1.6)	0.878	-	NS
Currently pregnant	94	15.9	1.6 (1.0-2.7)	0.067	-	NS
Hormonal contraceptives	230	33.6	0.6 (0.4-0.9)	**0.016**	-	NS
Intravaginal cleansing	132	21.9	1.0 (0.6-1.6)	0.841	-	NS
Visits bars	76	12.6	1.8 (1.0-3.1)	**0.027**	1.7 (0.96-3.1)	NS
Alcohol use	12	2	1.4 (0.4-5.1)	0.640	-	NS
Concurrent partners	85	14	1.1 (0.7-1.6)	0.799	-	NS
Condom use last act	207	34.3	1.2 (0.8-1.8)	0.487	-	NS
Partner >10 y older	202	33.4	1.2 (0.8-1.9)	0.335	-	NS
Experienced coercion**	169	28	0.7 (0.4-1.1)	0.142	0.6 (0.4-1.0)	0.058
Experienced force	38	6.3	1.3 (0.6-2.8)	0.541	-	NS
Sex for money or other benefits	10	1.7	1.3 (0.5-3.3)	0.579	-	NS

**VDS contains reported change in vaginal discharge, intermenstrual bleeding, or vaginal blood loss during or after sexual contact*

***Coercion reflects sex inequality in relationships, associated with increased risk*(17)

For peer review only

Determinants of *T. vaginalis* load

The median Cp value for vaginal *T. vaginalis* load of infection was 28.0 (range 17.0 – 37.9). We did not observe any significant associations between the *T. vaginalis* load and demographic factors (**Table 2**). Also no association was found between the *T. vaginalis* load and symptoms in general. However, in the group of women with *T. vaginalis* infection who had vaginal discharge observed during examination, a significantly higher load was observed as compared to those without vaginal discharge (P = 0.025).

Table 2. *T. vaginalis* load in association with risk factors

Risk factor		Median Cp	*P*
Age 18-24	18-24	28	0.747
	>24	27.7	
Single	Yes	27.3	0.971
	No	28	
Unemployed	Yes	27.7	0.810
	No	29.7	
HIV infected	Yes	27.5	0.986
	No	27.8	
History of VDS	Yes	28	0.954
	No	27.6	
Currently pregnant	Yes	28	0.794
	No	27.7	
Hormonal contraceptives	Yes	27.6	0.464
	No	28	
Intravaginal cleansing	Yes	28.5	0.433
	No	27.7	
Visits bars	Yes	25.3	0.310
	No	28	
Alcohol use	Yes	27.9	0.613
	No	30.2	
Concurrent partners	Yes	27	0.335
	No	28	
Condom use last sex act	Yes	27	0.339
	No	28	
Experiences force	Yes	29	0.603
	No	27.9	

For peer review only

DISCUSSION

In this study, we describe the prevalence, risk factors, and the load of *T. vaginalis* infection. This is one of a few studies to report these aspects for *T. vaginalis* in a cohort from South Africa. We observed a vaginal *T. vaginalis* prevalence of 20% (95% CI 17.0-23.4) and a rectal prevalence of 1.2% (95% CI 0.6-2.4).

In multivariate analysis two risk factors for vaginal *T. vaginalis* infection were identified: having a relationship status that is single and the presence of HIV infection. In addition, within women with vaginal *T. vaginalis* infection, a rectal *C. trachomatis* infection is significantly more present. Finally, a high *T. vaginalis* load appears to be associated with vaginal discharge observed during examination.

The prevalence of vaginal *T. vaginalis* infection in our study corresponds with the range reported by other studies in Africa (3.2%-40%)(14, 20). Rectal *T. vaginalis* infections are rarely described. One study from San Francisco, USA, describes a rectal *T. vaginalis* prevalence of 0.6%, based on 'men who have sex with men' (MSM) who reported receptive anal sex within six months prior to their clinic visit(21).

We show two demographic factors that may contribute to the high vaginal *T. vaginalis* prevalence in our study. First, we observed that a *T. vaginalis* infection is associated with a single relationship status. Joffe *et al.* described that a single marital status is often associated with having more than one sexual partner and thereby an increased risk for STI in general(22). To be more specific, it has been demonstrated that women having more than two concurrent male sexual partners are more likely to have a *T. vaginalis* infection(23). Although we were not able to confirm this, we hypothesize that a single marital status is associated with having more than one concurrent sexual partner.

It has been suggested that *T. vaginalis* infection are age-tied (24, 25). Women who were older than 20 years of age, were more likely to be infected with *T. vaginalis*(26). In our study we did not observe an association between age an T. vaginalis infections.

We confirm an association of positive HIV status with presence of *T. vaginalis* infection, which has been reported by several other studies(4, 8, 27-30). *T. vaginalis* infection was present in almost a quarter of HIV-infected women in our study. This is in line with the previously described higher trichomonas prevalence in high HIV prevalence cities compared to low HIV prevalence cities(14, 20). The biological link between HIV and TV is yet unclear, however bacterial vaginosis, is described to be an independent risk factor for acquisition of STI, and acquisition and transmission of HIV(31). Although we did not include bacterial vaginosis, it is described to be associated with a 1.5-2-fold increased risk for incident trichomonal infection(32). This could suggest an association between HIV and *T. vaginalis* where an asymptomatic *T. vaginalis* infection could increase the risk of HIV infection.

For peer review only

It has been described that non-sexual transmission of *T. vaginalis* occurs in girls who did not have sexual intercourse(33, 34). A striking prevalence found by a study among women from Ndola, Zambia, who denied ever had sexual intercourse was 43%(14). In those cases, *T. vaginalis* would be transmitted by wet clothes and water, since some studies suggest that *T. vaginalis* can survive up to days in warm mineral water and also for hours in urine(33). *T. vaginalis* is described to have some kind of robustness, which may explain its different transmission ways, compared to other STI. This may be an explanation of many hypotheses for the high prevalence, however, they need to be further explored.

Twenty-six percent of all women reported symptoms, without being diagnosed with a *T. vaginalis* infection. Although 20% of these women were diagnosed with another vaginal STI than *T. vaginalis*, symptoms are evidently a poor predictor for a *T. vaginalis* infection. We did not find a significant difference in symptomatology and *T. vaginalis* infection status. More knowledge about *T. vaginalis* disease would provide valuable information for potential targeted treatment approaches versus syndromic management.

In general, self-reported symptoms are not reliable in predicting an STI, like *T. vaginalis*(35, 36). However, 31 (16%) of the women vaginally infected with *T. vaginalis* were co-infected with either *C. trachomatis* or *N. gonorrhoeae* (data not shown). Symptoms of these frequent occurring STI may show comparable symptoms and should be taken into consideration. Although other studies revealed significant rates for co-infection between *T. vaginalis* and other STIs [12804936], in the current study we did not observe an increased risk for co-infection with *C. trachomatis* and/or *N. gonorrhoeae* in *T. vaginalis* infected patients (data not shown). However, in a previous study from our group we describe an increased risk of obtaining a *T. vaginalis* coinfection when infected with either *C. trachomatis* or *N. gonorrhoeae*. Concurrent STIs may be due to common risk factors for these infections, such as sexual activity, contraceptive use, and new partners.

This is one of a few studies to report aspects of rectal *T. vaginalis* in a cohort from South Africa. We observed that rectal *C. trachomatis* infections are significant more present in combination with a vaginal *T. vaginalis* infection. This may be due to several factors. First, sexual behaviour including anal sex is an important factor that may play a role in the presence of co-infections. Second, there is the ability to get re-infected via the gastrointestinal tract by auto inoculation, after being cured of the vaginal infection for Chlamydia, but it is unclear if this route of infection is also present for *T. vaginalis* infections and thus if the rectal site could be the reservoir for *T. vaginalis* infection(37). These potential transmission routes may result from vaginal wiping techniques, poor personal hygiene, using fingers during sex or for cleansing afterward, and use of sex toys(17). The most likely explanation is that the rectal samples may be contaminated by cervicovaginal fluid due to the close

For peer review only

anatomical proximity(38, 39) and that *T. vaginalis* positive rectal samples in women were contaminated with *T. vaginalis* positive vaginal fluid of the perianal surface(40).

It is conceivable that microorganism load may affect the clinical course of infection. It has been shown in studies of another STI, *C. trachomatis*, that there is an association between the bacterial load and genital manifestations of infection(41, 42). A higher *C. trachomatis* bacterial load is associated with the presence of at least two specific symptoms (vaginal discharge, irregular bleeding, or pelvic or abdominal pain). The impact of *T. vaginalis* load has not yet been described. We hypothesized that *T. vaginalis* load may affect the clinical course of the infection and compared several factors with the load of *T. vaginalis*. We did not find any significant association between the load and reported symptoms of a *T. vaginalis* infection. However, within women with observed vaginal discharge during examination, a significantly higher load was found. The load determination was performed using Cp values of the *T. vaginalis* PCR. Another technique to determine the average load per human is to incorporate the number of human cells. Our results prove that there is an association between *T. vaginalis* load and discharge. However, the literature on *T. vaginalis* load is scarce, and the effect of the load has to be further explored.

Although this is a unique study about the epidemiology and microbiological characteristics of *T. vaginalis* in rural South Africa with a relatively good sample size, a limitation of our study is that the number of rectal *T. vaginalis* infections is rather small for further analyses. As a consequence, this could have played a role in our statistical analyses. In addition, there can be a selection bias based on for instance the geographical inclusion, and self-reported symptoms can be more subjective than clinician based symptomatology. We cannot exclude potential cross-contamination between the cervico-vaginal and ano-rectal sites.

In conclusion, *T. vaginalis* infections are highly prevalent in the rural South African population investigated. The asymptomatic nature, potential for non-sexual transmission especially in socio-economic poor settings, and the syndromic management approach to STI control in this region are potential factors contributing to the prevalence. We observed a low rectal prevalence of which might be contamination with *T. vaginalis* positive vaginal fluid of the perianal surface. In multivariate analyses being single or HIV positive were identified as risk factors for vaginal *T. vaginalis* infection. Finally, a high *T. vaginalis* load seems associated with vaginal discharge observed during examination.

For peer review only

Funding

This work was supported by the Dutch Society for Tropical Medicine (NVTG), the Netherlands. The Anova Health Institute is supported by the US President's Emergency Plan for AIDS Relief program via the US Agency for International Development under Cooperative Agreement No. AID-674-A-12-00015. The views expressed in this manuscript do not necessarily reflect those of the President's Emergency Plan for AIDS Relief or the US Agency for International Development.

CONFLICT OF INTEREST

S.A.M. is a fulltime employee of the VU University Medical Center Amsterdam, and has been involved in the technical development of the Presto^{plus} CT-NG-TV assay (Microbiome, The Netherlands) via Microbiome Ltd. (S.A.M. is co-founder and co-director), a spin-off company of the VU University Medical Center, Amsterdam, the Netherlands.

None of the other authors report a potential conflict of interest.

For peer review only

1. Kissinger P. Epidemiology and treatment of trichomoniasis. Curr Infect Dis Rep. 2015;17(6):484.
2. World Health Organization DoRHaR. Global incidence and prevalence of selected curable sexually transmitted infections - 2008. 2012 2012. Report No.
3. Moodley P, Wilkinson D, Connolly C, Moodley J, Sturm AW. Trichomonas vaginalis is associated with pelvic inflammatory disease in women infected with human immunodeficiency virus. Clin Infect Dis. 2002;34(4):519-22.
4. Kissinger P, Adamski A. Trichomoniasis and HIV interactions: a review. Sex Transm Infect. 2013;89(6):426-33.
5. Cotch MF, Pastorek JG, Nugent RP, Hillier SL, Gibbs RS, Martin DH, et al. Trichomonas vaginalis associated with low birth weight and preterm delivery. The Vaginal Infections and Prematurity Study Group. Sex Transm Dis. 1997;24(6):353-60.
6. Soper D. Trichomoniasis: under control or undercontrolled? Am J Obstet Gynecol. 2004;190(1):281-90.
7. Van Der Pol B, Williams JA, Orr DP, Batteiger BE, Fortenberry JD. Prevalence, incidence, natural history, and response to treatment of Trichomonas vaginalis infection among adolescent women. J Infect Dis. 2005;192(12):2039-44.
8. Van Der Pol B. Trichomonas vaginalis infection: the most prevalent nonviral sexually transmitted infection receives the least public health attention. Clin Infect Dis. 2007;44(1):23-5.
9. Helms DJ, Mosure DJ, Metcalf CA, Douglas JM, Jr., Malotte CK, Paul SM, et al. Risk factors for prevalent and incident Trichomonas vaginalis among women attending three sexually transmitted disease clinics. Sex Transm Dis. 2008;35(5):484-8.
10. Schwebke JR, Hobbs MM, Taylor SN, Sena AC, Catania MG, Weinbaum BS, et al. Molecular testing for Trichomonas vaginalis in women: results from a prospective U.S. clinical trial. J Clin Microbiol. 2011;49(12):4106-11.
11. van Der Schee C, van Belkum A, Zwijgers L, van Der Brugge E, O'Neill E L, Luijendijk A, et al. Improved diagnosis of Trichomonas vaginalis infection by PCR using vaginal swabs and urine specimens compared to diagnosis by wet mount microscopy, culture, and fluorescent staining. J Clin Microbiol. 1999;37(12):4127-30.
12. Sena AC, Miller WC, Hobbs MM, Schwebke JR, Leone PA, Swygard H, et al. Trichomonas vaginalis infection in male sexual partners: implications for diagnosis, treatment, and prevention. Clin Infect Dis. 2007;44(1):13-22.
13. Vatanshenassan M, Rezaie S, Mohebali M, Niromand N, Kazemi B, Babaei Z, et al. Trichomonas vaginalis: investigation of a novel diagnostic method in urine samples using cysteine proteinase 4 gene and PCR technique. Exp Parasitol. 2010;126(2):187-90.
14. Buve A, Weiss HA, Laga M, Van DE, Musonda R, Zekeng L, et al. The epidemiology of trichomoniasis in women in four African cities. AIDS. 2001;15 Suppl 4:S89-S96.
15. Michel CE, Sonnex C, Carne CA, White JA, Magbanua JP, Nadala EC, Jr., et al. Chlamydia trachomatis load at matched anatomic sites: implications for screening strategies. J Clin Microbiol. 2007;45(5):1395-402.
16. Bissessor M, Tabrizi SN, Fairley CK, Danielewski J, Whitton B, Bird S, et al. Differing Neisseria gonorrhoeae bacterial loads in the pharynx and rectum in men who have sex with men: implications for gonococcal detection, transmission, and control. J Clin Microbiol. 2011;49(12):4304-6.
17. Peters RP, Dubbink JH, van der Eem L, Verweij SP, Bos ML, Ouburg S, et al. Cross-sectional study of genital, rectal, and pharyngeal Chlamydia and gonorrhea in women in rural South Africa. Sex Transm Dis. 2014;41(9):564-9.

For peer review only

18. de Waaij DJ, Ouburg S, Dubbink JH, Peters RP, Morre SA. Evaluation of Presto(plus) assay and LightMix kit Trichomonas vaginalis assay for detection of Trichomonas vaginalis in dry vaginal swabs. J Microbiol Methods. 2016;127:102-4.
19. Hoffman S, O'Sullivan LF, Harrison A, Dolezal C, Monroe-Wise A. HIV risk behaviors and the context of sexual coercion in young adults' sexual interactions: results from a diary study in rural South Africa. Sex Transm Dis. 2006;33(1):52-8.
20. Naidoo S, Wand H. Prevalence and incidence of Trichomonas vaginalis infections in women participating in a clinical trial in Durban, South Africa. Sex Transm Infect. 2013;89(6):519-22.
21. Dubbink JH, de Waaij DJ, Bos M, van der Eem L, Bebear C, Mbambazela N, et al. Microbiological Characteristics of Chlamydia trachomatis and Neisseria gonorrhoeae Infections in South African Women. J Clin Microbiol. 2016;54(1):200-3.
22. Joffe GP, Foxman B, Schmidt AJ, Farris KB, Carter RJ, Neumann S, et al. Multiple partners and partner choice as risk factors for sexually transmitted disease among female college students. Sex Transm Dis. 1992;19(5):272-8.
23. Miller M, Liao Y, Gomez AM, Gaydos CA, D'Mellow D. Factors associated with the prevalence and incidence of Trichomonas vaginalis infection among African American women in New York city who use drugs. J Infect Dis. 2008;197(4):503-9.
24. Ginocchio CC, Chapin K, Smith JS, Aslanzadeh J, Snook J, Hill CS, et al. Prevalence of Trichomonas vaginalis and coinfection with Chlamydia trachomatis and Neisseria gonorrhoeae in the United States as determined by the Aptima Trichomonas vaginalis nucleic acid amplification assay. J Clin Microbiol. 2012;50(8):2601-8.
25. Munson E, Kramme T, Napierala M, Munson KL, Miller C, Hryciuk JE. Female epidemiology of transcription-mediated amplification-based Trichomonas vaginalis detection in a metropolitan setting with a high prevalence of sexually transmitted infection. J Clin Microbiol. 2012;50(12):3927-31.
26. Kwena Z, Sharma A, Wamae N, Muga C, Bukusi E. Provider characteristics among staff providing care to sexually transmitted infection self-medicating patients in retail pharmacies in Kibera slum, Nairobi, Kenya. Sex Transm Dis. 2008;35(5):480-3.
27. Sorvillo F, Smith L, Kerndt P, Ash L. Trichomonas vaginalis, HIV, and African-Americans. Emerg Infect Dis. 2001;7(6):927-32.
28. Chun HM, Carpenter RJ, Macalino GE, Crum-Cianflone NF. The Role of Sexually Transmitted Infections in HIV-1 Progression: A Comprehensive Review of the Literature. J Sex Transm Dis. 2013;2013:176459.
29. Fastring DR, Amedee A, Gatski M, Clark RA, Mena LA, Levison J, et al. Co-occurrence of Trichomonas vaginalis and bacterial vaginosis and vaginal shedding of HIV-1 RNA. Sex Transm Dis. 2014;41(3):173-9.
30. Fowke JH, Han X, Alderete JF, Moses KA, Signorello LB, Blot WJ. A prospective study of Trichomonas vaginalis and prostate cancer risk among African American men. BMC Res Notes. 2016;9:224.
31. Brotman RM. Vaginal microbiome and sexually transmitted infections: an epidemiologic perspective. J Clin Invest. 2011;121(12):4610-7.
32. Brotman RM, Klebanoff MA, Nansel TR, Yu KF, Andrews WW, Zhang J, et al. Bacterial vaginosis assessed by gram stain and diminished colonization resistance to incident gonococcal, chlamydial, and trichomonal genital infection. J Infect Dis. 2010;202(12):1907-15.
33. Neinstein LS, Goldenring J, Carpenter S. Nonsexual transmission of sexually transmitted diseases: an infrequent occurrence. Pediatrics. 1984;74(1):67-76.

For peer review only

34. Crucitti T, Jespers V, Mulenga C, Khondowe S, Vandepitte J, Buve A. Non-sexual transmission of Trichomonas vaginalis in adolescent girls attending school in Ndola, Zambia. PLoS One. 2011;6(1):e16310.
35. Nagarajan K, S G, Ramakrishnan L, Mainkar M, Ramesh S, Ganju D, et al. Self-Report of STI Symptoms, Inconsistent Condom use and Condom Nonuse are Poor Predictors of STI Prevalence among Men who have Sex with Men. Journal of AIDS & Clinical Research. 2013.
36. van der Eem L, Dubbink JH, Struthers HE, McIntyre JA, Ouburg S, Morre SA, et al. Evaluation of syndromic management guidelines for treatment of sexually transmitted infections in South African women. Trop Med Int Health. 2016;21(9):1138-46.
37. Rank RG, Yeruva L. Hidden in plain sight: chlamydial gastrointestinal infection and its relevance to persistence in human genital infection. Infect Immun. 2014;82(4):1362-71.
38. Francis SC, Kent CK, Klausner JD, Rauch L, Kohn R, Hardick A, et al. Prevalence of rectal Trichomonas vaginalis and Mycoplasma genitalium in male patients at the San Francisco STD clinic, 2005-2006. Sex Transm Dis. 2008;35(9):797-800.
39. Crucitti T, Jespers V, Mulenga C, Khondowe S, Vandepitte J, Buve A. Trichomonas vaginalis is highly prevalent in adolescent girls, pregnant women, and commercial sex workers in Ndola, Zambia. Sex Transm Dis. 2010;37(4):223-7.
40. Cosentino LA, Campbell T, Jett A, Macio I, Zamborsky T, Cranston RD, et al. Use of nucleic acid amplification testing for diagnosis of anorectal sexually transmitted infections. J Clin Microbiol. 2012;50(6):2005-8.
41. Jalal H, Verlander NQ, Kumar N, Bentley N, Carne C, Sonnex C. Genital chlamydial infection: association between clinical features, organism genotype and load. J Med Microbiol. 2011;60(Pt 7):881-8.
42. Gomes JP, Borrego MJ, Atik B, Santo I, Azevedo J, Brito de SA, et al. Correlating Chlamydia trachomatis infectious load with urogenital ecological success and disease pathogenesis. Microbes Infect. 2006;8(1):16-26.

For peer review only

BMJ Open

Prevalence of Trichomonas vaginalis infection and protozoan load in South African women

Journal:	*BMJ Open*
Manuscript ID	bmjopen-2017-016959.R2
Article Type:	Research
Date Submitted by the Author:	26-Jul-2017

Complete List of Authors:	de Waaij, Dewi; VU University Medical Centre, Laboratory of Immunogenetics, Dept. Medical Microbiology and Infection Control<br>Dubbink, Jan Henk; Anova Health Institute; University of Maastricht, Institute for Public Health Genomics, Faculty of Health, Medicine and LIfe Sciences<br>Ouburg, Sander; VU University Medical Centre, Laboratory of Immunogenetics, Dept. Medical Microbiology and Infection Control<br>Peters, Remco; Anova Health Institute, ; Maastricht University Medical Centre, Medical Microbiology<br>Morre, S; VU University medical center, Pathology, Laboratory of Immunogenetics

**Primary Subject Heading**:	Infectious diseases
Secondary Subject Heading:	Epidemiology, Infectious diseases, Sexual health
Keywords:	EPIDEMIOLOGY, INFECTIOUS DISEASES, Molecular diagnostics < INFECTIOUS DISEASES

SCHOLARONE™
Manuscripts

Prevalence of *Trichomonas vaginalis* infection and protozoan load in South African women

Dewi J. de Waaij[1,2] , Jan Henk Dubbink[1,2], Sander Ouburg[1*], Remco P.H. Peters[3,4], Servaas A. Morré[1,2]

1 VU University Medical Centre, Department of Medical Microbiology & Infection Control, Laboratory of Immunogenetics, Amsterdam, The Netherlands.

2 Institute for Public Health Genomics (IPHG), Department of Genetics and Cell Biology, Research School GROW (School for Oncology & Developmental Biology), Faculty of Health, Medicine & Life Sciences, University of Maastricht, Maastricht, The Netherlands.

3 Anova Health Institute, Johannesburg and Tzaneen, South Africa.

4 Department of Medical Microbiology, University of Maastricht, Maastricht, The Netherlands.

* Corresponding author

dr. S. Ouburg, VU University Medical Center, Laboratory of Immunogenetics, dept. Medical Microbiology and Infection Control, De Boelelaan 1108, 1081HZ Amsterdam, the Netherlands. Tel.: +31 (0)20 444 8417, Fax.: +31 (0)20 444 8418. E-mail: s.ouburg@vumc.nl

Keywords: **Trichomonas vaginalis, risk factors, protozoan load, microbiological characteristics, rural South Africa**

Word count: 2841

Journal: BMJ open

For peer review only

ABSTRACT

Objectives. *Trichomonas vaginalis* is thought to be the most common non-viral sexually transmitted infection (STI) worldwide. We investigated the prevalence, risk factors and protozoan load of *Trichomonas vaginalis* (*T. vaginalis* load) infection in South African women.

Methods. A cross-sectional study of 604 women was conducted at 25 primary healthcare facilities in rural South Africa (Mopani district). *T. vaginalis* DNA was detected in vaginal and rectal swabs. In a uni- and multivariate analyses the *T. vaginalis* infection was investigated in relation to demographic characteristics, medical history, and behavioural factors. The *T. vaginalis* load was determined as the logarithm of DNA copies per microliter sample solution.

Results. Collected vaginal and rectal swabs were tested for *T. vaginalis* DNA. Prevalence of vaginal *T. vaginalis* was 20% (95% CI 17.0-23.4) and rectal 1.2% (95% CI 0.6-2.4). Most women (66%) with a vaginal infection were asymptomatic. Factors associated with *T. vaginalis* infection were a relationship status of single (OR, 2.4; 95% CI, 1.5-4.0; $P < 0.001$) and HIV positive-infection (OR, 1.6; 95% CI, 1.0-2.6; $P = 0.041$). Women with vaginal *T. vaginalis* infection were more likely to have concurrent *C. trachomatis* rectal infection than those without vaginal infection (12% vs. 3%; $p<0.001$, OR: 4.1). A higher median *T. vaginalis* load was observed among women with observed vaginal discharge compared to those without vaginal discharge ($P = 0.025$).

Conclusions. Vaginal trichomoniasis is highly prevalent in rural South Africa, especially among single women and those with HIV infection, and often presents without symptoms.

Strengths and limitations of this study

- This is the first study about the epidemiology and microbiological characteristics of *T. vaginalis* in rural South Africa
- The study has a high sample size
- The number of rectal *T. vaginalis* infections is relatively low
- A selection bias is possible based on the geographical inclusion

Data sharing: All data of this study have been described in the paper. No additional data for this study are available.

For peer review only

Author contributions:

DJdW: Laboratory analyses, statistical analyses, writing of the manuscript

JHD: Sample collection, writing of the manuscript

SO: Statistical analyses, reviewing of the manuscript

RPHP: Sample collection, reviewing of the manuscript, study supervision

SAM: reviewing of the manuscript, study supervision.

For peer review only

INTRODUCTION

Trichomonas vaginalis is a protozoan parasite that causes trichomoniasis and is mostly sexually transmitted. It is the most common non-viral sexually transmitted infection (STI) worldwide (1). The infection is asymptomatic in 85% of the women and in 77% of the men(1). The WHO describes that *T. vaginalis* in the African region occurs ten times as often in women than in men(2). Vaginal *T. vaginalis* infections in the entire African region is estimated to be 42.8 million(2). If the infection is symptomatic, women may report a change in vaginal discharge, intermenstrual bleeding, or vaginal blood loss during or after sexual contact. Other symptoms that can occur are vaginal itching, dysuria, and abdominal pain. Also upper reproductive tract disease syndromes can occur, including pelvic inflammatory disease. Women with a *T. vaginalis* infection have 4.7-fold increase in the risk of PID(3-5) and tubal pathology(6). Women infected with *T. vaginalis* also have a 1.3-fold increase in the risk of preterm labour(5).

In women, *T. vaginalis* infection can persist for months and, if left untreated, it can increase the risk for HIV acquisition if exposed(6, 7). *T. vaginalis* could lead to an increase in the vaginal HIV load and thus potentially increase the risk of HIV transmission to a sexual partner(8). In addition, an association between *T. vaginalis* and *Chlamydia trachomatis* infection has been found(9). Having a concurrent chlamydial infection was a predictor of both prevalent and incident *T. vaginalis*.

Despite the estimated large burden of *T. vaginalis* infection in the African region, data on clinical presentation, demographic and behavioural factors associated with infection, and microbiological factors is relatively limited. However, in other continents, multiple studies have been done in the context of *T. vaginalis* infections(10-13). *T. vaginalis* prevalence has been reported for a few African countries and ranges from 6.5-40%(14). One of the explanations for the often high *T. vaginalis* prevalence is the lack of STI screening programmes and limited control measurements. The estimated burden of disease is significant but available data of symptomatology, co-infections and pathogen load are limited. *C. trachomatis* and *Neisseria gonorrhoeae* are other important causative micro-organisms for STI, showing an association with the pathogen load as compared to the clinical presentation and the development of long-term sequelae(15, 16).

In this study, we present the epidemiology of *T. vaginalis* infection including the *T. vaginalis* load, as measured by a validated nucleic acid amplification technique. We studied a high HIV prevalence setting in rural South Africa.

For peer review only

METHODS

Population and Design

This study was part of a cross-sectional study of 604 women in rural Mopani District, South Africa, as described previously(17). In brief, women aged 18-49 years old who reported sexual activity during the prior 6 months were recruited at 25 primary healthcare (PHC) facilities, regardless of the reason for visiting the facility that day. After informed consent, demographic characteristics, medical history and details of sexual behaviour were collected using a questionnaire. Healthcare worker collected vaginal and rectal swabs were obtained for molecular testing of *T. vaginalis*.

The Human Ethics Research Committee of the University of the Witwatersrand, South Africa, approved the study (Ref M110726).

Laboratory testing

Collected vaginal and rectal swabs were analysed at the laboratory of Medical Microbiology & Infection Control, VU University Medical Centre, Amsterdam, the Netherlands. High Pure PCR Template Preparation Kit (HPPTP) (Roche Diagnostics, Basel, Switzerland) was used for the extraction of bacterial DNA. Detection of *T. vaginalis* DNA was done with the Presto^{plus} assay (Microbiome Ltd., Amsterdam, the Netherlands) and the LightCycler II 480 (Roche Diagnostics) was used for the quantitative PCR for *C. trachomatis*, *N. gonorrhoeae* and *T. vaginalis*(18). The Presto^{plus} assay for *C. trachomatis, N. gonorrhoeae,* and *T. vaginalis* is currently for in house use only and it will be transformed into a Presto-TV (Goffin Molecular Technologies, The Netherlands) in 2017 in a 200 reaction format as CE-IVD certified kit. PCR positivity was determined using the Cp (crossing point) value: we used a threshold Cp of <38 for a sample to be classified as positive. Samples with Cp values of ≥38 were retested and if again ≥38 were excluded from further analyses (indeterminate samples).

Data analyses

The following risk factors were used in our analyses: age, being single, unemployment, current pregnancy, history of vaginal discharge syndrome (VDS), currently being pregnant, the use of hormonal contraceptives, administering intravaginal cleansing, visiting bars, alcohol use, concurrent partners, condom use during last sex act, partner >10 years older, experiencing coercion and/or force, and sex for money or for other benefits(17). Coercion reflects sex inequality in relationships which is associated with increased risk(19).

For peer review only

Data were double-entered in Epi Data (Epi Info™ version 3.5.3) by two researchers independently, were compared for inconsistencies, cleaned and verified. The data were analysed using SPSS version 20.0 (SPSS Inc., Chicago, USA). Dichotomous data were compared using χ^2 test or Fisher's Exact test and continuous data using the Mann-Whitney *U* test. Odds ratios (ORs) with 95% confidence interval (CI) were calculated. Vaginal *T. vaginalis* infection was defined symptomatic when either abnormal vaginal discharge during physical examination, intermenstrual bleeding, or vaginal blood loss during or after sexual intercourse was reported. Univariate analysis was performed to examine possible associations of demographic variables and clinical and behavioural factors in relation to *T. vaginalis*. Multivariate analysis was conducted using a backward selection procedure and likelihood ratio tests. As a proxy for the *T. vaginalis* load we used the median Cp value, which was calculated for each risk factor and compared between infected versus non-infected women.

For peer review only

RESULTS

Demographics of study population

A quarter of all women (n= 154; 25.5%) had an age 18-24, over half of the women were single (334; (55.3%), 26 women reported anal intercourse (4.3%), a high proportion was unemployed, and almost 30% reported to be infected with HIV (177; 29.3%). For more details see **Table 1**.

Prevalence of *T. vaginalis* infection

Of the 604 vaginal samples, one sample was excluded based on lack of biological material and 28 gave an indeterminate result (2 times Cp values of ≥38); these were excluded resulting in 575 samples for analysis. For vaginal infection, 113/575 (20%) samples tested positive.

Of the 604 rectal samples, nine samples gave an indeterminate result (2 times Cp values of ≥38) and were excluded resulting in 595 samples. For rectal infection, 7/595 (1.2%) samples tested positive. Four patients were infected *T. vaginalis* at both the rectal and vaginal site.

Clinical manifestation of infection

Symptoms of vaginal infection were reported by 196 (34%) of the women. Of these, 38 had *T. vaginalis* infection meaning 38/113 (34%) of the women with TV reported symptoms. Of the 462 women without *T. vaginalis,* 158 reported symptoms of infection (34%). None of the individual symptoms (vaginal discharge, intermenstrual bleeding and vaginal blood loss related to sexual contact) was associated with *T. vaginalis* infection. For rectal infection (n=7) the numbers were low and no rectal symptomatology was scored.

Factors associated with *Trichomonas vaginalis* infection

In univariate analyses we observed significant associations for single status, being HIV positive, the use of hormonal contraceptives, and visiting bars (**Table 1**). In multivariate analysis (**Table 1**), having a relationship status that is single was significantly associated with a vaginal *T. vaginalis* infection (adjusted OR [aOR], 2.4 [95% CI, 1.5-4.0, p <0.001]) and presence of HIV infection was significantly associated (aOR, 1.6; [95% CI 1.0-2.6; p = 0.041]) *T. vaginalis* infection. Within women with a current vaginal *T. vaginalis* infection, a rectal *C. trachomatis* infection is significantly more present (P<0.001, OR: 4.1).

For peer review only

188

189 **Table 1.** Univariate and Multivariate Analysis of Factors associated with Vaginal *Trichomonas vaginalis* Infection
190 in South African Women

			Univariate		Multivariate	
Factor	n	%	OR (95% CI)	*P*	OR (95% CI)	*P*
Age 18-24	154	25.5	0.9 (0.6-1.6)	0.95	-	NS
Single	334	55.3	2.4 (1.5-3.9)	**<0.001**	2.4 (1.5-4.0)	**<0.001**
Unemployed	424	70.2	1.5 (0.9-2.4)	0.135	-	NS
HIV infected	177	29.3	1.6 (1.0-2.4)	**0.040**	1.6 (1.0-2.6)	**0.041**
History of VDS*	196	32.4	1.0 (0.7-1.6)	0.878	-	NS
Currently pregnant	94	15.9	1.6 (1.0-2.7)	0.067	-	NS
Hormonal contraceptives	230	33.6	0.6 (0.4-0.9)	**0.016**	-	NS
Intravaginal cleansing	132	21.9	1.0 (0.6-1.6)	0.841	-	NS
Visits bars	76	12.6	1.8 (1.0-3.1)	**0.027**	1.7 (0.96-3.1)	NS
Alcohol use	12	2	1.4 (0.4-5.1)	0.640	-	NS
Concurrent partners	85	14	1.1 (0.7-1.6)	0.799	-	NS
Condom use last act	207	34.3	1.2 (0.8-1.8)	0.487	-	NS
Partner >10 y older	202	33.4	1.2 (0.8-1.9)	0.335	-	NS
Experienced coercion**	169	28	0.7 (0.4-1.1)	0.142	0.6 (0.4-1.0)	0.058
Experienced force	38	6.3	1.3 (0.6-2.8)	0.541	-	NS
Sex for money or other benefits	10	1.7	1.3 (0.5-3.3)	0.579	-	NS

191 **VDS contains reported change in vaginal discharge, intermenstrual bleeding, or vaginal blood loss during or after sexual*
192 *contact*
193 ***Coercion reflects sex inequality in relationships, associated with increased risk*(17)
194

195

196

197

For peer review only

Determinants of *T. vaginalis* load

The median Cp value for vaginal *T. vaginalis* load of infection was 28.0 (range 17.0 – 37.9). We did not observe any significant associations between the *T. vaginalis* load and demographic factors (**Table 2**). Also no association was found between the *T. vaginalis* load and symptoms in general. However, in the group of women with *T. vaginalis* infection who had vaginal discharge observed during examination, a significantly higher load was observed as compared to those without vaginal discharge (P = 0.025).

Table 2. *T. vaginalis* load in association with risk factors

Risk factor		Median Cp	*P*
Age 18-24	18-24	28	0.747
	>24	27.7	
Single	Yes	27.3	0.971
	No	28	
Unemployed	Yes	27.7	0.810
	No	29.7	
HIV infected	Yes	27.5	0.986
	No	27.8	
History of VDS	Yes	28	0.954
	No	27.6	
Currently pregnant	Yes	28	0.794
	No	27.7	
Hormonal contraceptives	Yes	27.6	0.464
	No	28	
Intravaginal cleansing	Yes	28.5	0.433
	No	27.7	
Visits bars	Yes	25.3	0.310
	No	28	
Alcohol use	Yes	27.9	0.613
	No	30.2	
Concurrent partners	Yes	27	0.335
	No	28	
Condom use last sex act	Yes	27	0.339
	No	28	
Experiences force	Yes	29	0.603
	No	27.9	

For peer review only

For peer review only - http://bmjopen.bmj.com/site/about/guidelines.xhtml

DISCUSSION

In this study, we describe the prevalence, risk factors, and the load of *T. vaginalis* infection. This is one of a few studies to report these aspects for *T. vaginalis* in a cohort from South Africa. We observed a vaginal *T. vaginalis* prevalence of 20% (95% CI 17.0-23.4) and a rectal prevalence of 1.2% (95% CI 0.6-2.4).

In multivariate analysis two risk factors for vaginal *T. vaginalis* infection were identified: having a relationship status that is single and the presence of HIV infection. In addition, within women with vaginal *T. vaginalis* infection, a rectal *C. trachomatis* infection is significantly more present. Finally, a high *T. vaginalis* load appears to be associated with vaginal discharge observed during examination.

The prevalence of vaginal *T. vaginalis* infection in our study corresponds with the range reported by other studies in Africa (3.2%-40%)(14, 20). Rectal *T. vaginalis* infections are rarely described. One study from San Francisco, USA, describes a rectal *T. vaginalis* prevalence of 0.6%, based on 'men who have sex with men' (MSM) who reported receptive anal sex within six months prior to their clinic visit(21, 22).

We show two demographic factors that may contribute to the high vaginal *T. vaginalis* prevalence in our study. First, we observed that a *T. vaginalis* infection is associated with a single relationship status. Joffe *et al.* described that a single marital status is often associated with having more than one sexual partner and thereby an increased risk for STI in general(23). To be more specific, it has been demonstrated that women having more than two concurrent male sexual partners are more likely to have a *T. vaginalis* infection(24). Although we were not able to confirm this, we hypothesize that a single marital status is associated with having more than one concurrent sexual partner.

It has been suggested that *T. vaginalis* infection are age-tied(25, 26). Women who were older than 20 years of age, were more likely to be infected with *T. vaginalis*(27). In our study we did not observe an association between age an T. vaginalis infections.

We confirm an association of positive HIV status with presence of *T. vaginalis* infection, which has been reported by several other studies(4, 8, 28-31). *T. vaginalis* infection was present in almost a quarter of HIV-infected women in our study. This is in line with the previously described higher trichomonas prevalence in high HIV prevalence cities compared to low HIV prevalence cities(14, 20). The biological link between HIV and TV is yet unclear, however bacterial vaginosis, is described to be an independent risk factor for acquisition of STI, and acquisition and transmission of HIV(32). Although we did not include bacterial vaginosis, it is described to be associated with a 1.5-2-fold increased risk for incident trichomonal infection(33). This could suggest an association between HIV and *T. vaginalis* where an asymptomatic *T. vaginalis* infection could increase the risk of HIV infection.

It has been described that non-sexual transmission of *T. vaginalis* occurs in girls who did not have sexual intercourse(34, 35). A striking prevalence found by a study among women from Ndola, Zambia, who denied ever had sexual intercourse was 43%(14). In those cases, *T. vaginalis* would be transmitted by wet clothes and water, since some studies suggest that *T. vaginalis* can survive up to days in warm mineral water and also for hours in urine(34). *T. vaginalis* is described to have some kind of robustness, which may explain its different transmission ways, compared to other STI. This may be an explanation of many hypotheses for the high prevalence, however, they need to be further explored.

Twenty-six percent of all women reported symptoms, without being diagnosed with a *T. vaginalis* infection. Although 20% of these women were diagnosed with another vaginal STI than *T. vaginalis*, symptoms are evidently a poor predictor for a *T. vaginalis* infection. We did not find a significant difference in symptomatology and *T. vaginalis* infection status. More knowledge about *T. vaginalis* disease would provide valuable information for potential targeted treatment approaches versus syndromic management.

In general, self-reported symptoms are not reliable in predicting an STI, like *T. vaginalis*(36, 37). However, 31 (16%) of the women vaginally infected with *T. vaginalis* were co-infected with either *C. trachomatis* or *N. gonorrhoeae* (data not shown). Symptoms of these frequent occurring STI may show comparable symptoms and should be taken into consideration. Although other studies revealed significant rates for co-infection between *T. vaginalis* and other STIs [12804936], in the current study we did not observe an increased risk for co-infection with *C. trachomatis* and/or *N. gonorrhoeae* in *T. vaginalis* infected patients (data not shown). However, in a previous study from our group we describe an increased risk of obtaining a *T. vaginalis* coinfection when infected with either *C. trachomatis* or *N. gonorrhoeae*. Concurrent STIs may be due to common risk factors for these infections, such as sexual activity, contraceptive use, and new partners.

This is one of a few studies to report aspects of rectal *T. vaginalis* in a cohort from South Africa. We observed that rectal *C. trachomatis* infections are significant more present in combination with a vaginal *T. vaginalis* infection. This may be due to several factors. First, sexual behaviour including anal sex is an important factor that may play a role in the presence of co-infections. Second, there is the ability to get re-infected via the gastrointestinal tract by auto inoculation, after being cured of the vaginal infection for Chlamydia, but it is unclear if this route of infection is also present for *T. vaginalis* infections and thus if the rectal site could be the reservoir for *T. vaginalis* infection(38). These potential transmission routes may result from vaginal wiping techniques, poor personal hygiene, using fingers during sex or for cleansing afterward, and use of sex toys(17). The most likely explanation is that the rectal samples may be contaminated by cervicovaginal fluid due to the close

For peer review only

anatomical proximity(39, 40) and that *T. vaginalis* positive rectal samples in women were contaminated with *T. vaginalis* positive vaginal fluid of the perianal surface(41).

It is conceivable that microorganism load may affect the clinical course of infection. It has been shown in studies of another STI, *C. trachomatis*, that there is an association between the bacterial load and genital manifestations of infection(42, 43). A higher *C. trachomatis* bacterial load is associated with the presence of at least two specific symptoms (vaginal discharge, irregular bleeding, or pelvic or abdominal pain). The impact of *T. vaginalis* load has not yet been described. We hypothesized that *T. vaginalis* load may affect the clinical course of the infection and compared several factors with the load of *T. vaginalis*. We did not find any significant association between the load and reported symptoms of a *T. vaginalis* infection. However, within women with observed vaginal discharge during examination, a significantly higher load was found. The load determination was performed using Cp values of the *T. vaginalis* PCR. Another technique to determine the average load per human is to incorporate the number of human cells. Our results prove that there is an association between *T. vaginalis* load and discharge. However, the literature on *T. vaginalis* load is scarce, and the effect of the load has to be further explored.

Although this is a unique study about the epidemiology and microbiological characteristics of *T. vaginalis* in rural South Africa with a relatively good sample size, a limitation of our study is that the number of rectal *T. vaginalis* infections is rather small for further analyses. As a consequence, this could have played a role in our statistical analyses. In addition, there can be a selection bias based on for instance the geographical inclusion, and self-reported symptoms can be more subjective than clinician based symptomatology. We cannot exclude potential cross-contamination between the cervico-vaginal and ano-rectal sites.

In conclusion, *T. vaginalis* infections are highly prevalent in the rural South African population investigated. The asymptomatic nature, potential for non-sexual transmission especially in socio-economic poor settings, and the syndromic management approach to STI control in this region are potential factors contributing to the prevalence. We observed a low rectal prevalence of which might be contamination with *T. vaginalis* positive vaginal fluid of the perianal surface. In multivariate analyses being single or HIV positive were identified as risk factors for vaginal *T. vaginalis* infection. Finally, a high *T. vaginalis* load seems associated with vaginal discharge observed during examination.

For peer review only

Funding

This work was supported by the Dutch Society for Tropical Medicine (NVTG), the Netherlands. The Anova Health Institute is supported by the US President's Emergency Plan for AIDS Relief program via the US Agency for International Development under Cooperative Agreement No. AID-674-A-12-00015. The views expressed in this manuscript do not necessarily reflect those of the President's Emergency Plan for AIDS Relief or the US Agency for International Development.

CONFLICT OF INTEREST

S.A.M. is a fulltime employee of the VU University Medical Center Amsterdam, and has been involved in the technical development of the Presto^{plus} CT-NG-TV assay (Microbiome, The Netherlands) via Microbiome Ltd. (S.A.M. is co-founder and co-director), a spin-off company of the VU University Medical Center, Amsterdam, the Netherlands.

None of the other authors report a potential conflict of interest.

For peer review only

1. Kissinger P. Epidemiology and treatment of trichomoniasis. Curr Infect Dis Rep. 2015;17(6):484.
2. World Health Organization DoRHaR. Global incidence and prevalence of selected curable sexually transmitted infections - 2008. 2012 2012. Report No.
3. Moodley P, Wilkinson D, Connolly C, Moodley J, Sturm AW. Trichomonas vaginalis is associated with pelvic inflammatory disease in women infected with human immunodeficiency virus. Clin Infect Dis. 2002;34(4):519-22.
4. Kissinger P, Adamski A. Trichomoniasis and HIV interactions: a review. Sex Transm Infect. 2013;89(6):426-33.
5. Cotch MF, Pastorek JG, Nugent RP, Hillier SL, Gibbs RS, Martin DH, et al. Trichomonas vaginalis associated with low birth weight and preterm delivery. The Vaginal Infections and Prematurity Study Group. Sex Transm Dis. 1997;24(6):353-60.
6. Soper D. Trichomoniasis: under control or undercontrolled? Am J Obstet Gynecol. 2004;190(1):281-90.
7. Van Der Pol B, Williams JA, Orr DP, Batteiger BE, Fortenberry JD. Prevalence, incidence, natural history, and response to treatment of Trichomonas vaginalis infection among adolescent women. J Infect Dis. 2005;192(12):2039-44.
8. Van Der Pol B. Trichomonas vaginalis infection: the most prevalent nonviral sexually transmitted infection receives the least public health attention. Clin Infect Dis. 2007;44(1):23-5.
9. Helms DJ, Mosure DJ, Metcalf CA, Douglas JM, Jr., Malotte CK, Paul SM, et al. Risk factors for prevalent and incident Trichomonas vaginalis among women attending three sexually transmitted disease clinics. Sex Transm Dis. 2008;35(5):484-8.
10. Schwebke JR, Hobbs MM, Taylor SN, Sena AC, Catania MG, Weinbaum BS, et al. Molecular testing for Trichomonas vaginalis in women: results from a prospective U.S. clinical trial. J Clin Microbiol. 2011;49(12):4106-11.
11. van Der Schee C, van Belkum A, Zwijgers L, van Der Brugge E, O'Neill E L, Luijendijk A, et al. Improved diagnosis of Trichomonas vaginalis infection by PCR using vaginal swabs and urine specimens compared to diagnosis by wet mount microscopy, culture, and fluorescent staining. J Clin Microbiol. 1999;37(12):4127-30.
12. Sena AC, Miller WC, Hobbs MM, Schwebke JR, Leone PA, Swygard H, et al. Trichomonas vaginalis infection in male sexual partners: implications for diagnosis, treatment, and prevention. Clin Infect Dis. 2007;44(1):13-22.
13. Vatanshenassan M, Rezaie S, Mohebali M, Niromand N, Kazemi B, Babaei Z, et al. Trichomonas vaginalis: investigation of a novel diagnostic method in urine samples using cysteine proteinase 4 gene and PCR technique. Exp Parasitol. 2010;126(2):187-90.
14. Buve A, Weiss HA, Laga M, Van DE, Musonda R, Zekeng L, et al. The epidemiology of trichomoniasis in women in four African cities. AIDS. 2001;15 Suppl 4:S89-S96.
15. Michel CE, Sonnex C, Carne CA, White JA, Magbanua JP, Nadala EC, Jr., et al. Chlamydia trachomatis load at matched anatomic sites: implications for screening strategies. J Clin Microbiol. 2007;45(5):1395-402.
16. Bissessor M, Tabrizi SN, Fairley CK, Danielewski J, Whitton B, Bird S, et al. Differing Neisseria gonorrhoeae bacterial loads in the pharynx and rectum in men who have sex with men: implications for gonococcal detection, transmission, and control. J Clin Microbiol. 2011;49(12):4304-6.
17. Peters RP, Dubbink JH, van der Eem L, Verweij SP, Bos ML, Ouburg S, et al. Cross-sectional study of genital, rectal, and pharyngeal Chlamydia and gonorrhea in women in rural South Africa. Sex Transm Dis. 2014;41(9):564-9.

For peer review only - http://bmjopen.bmj.com/site/about/guidelines.xhtml

18. de Waaij DJ, Ouburg S, Dubbink JH, Peters RP, Morre SA. Evaluation of Presto(plus) assay and LightMix kit Trichomonas vaginalis assay for detection of Trichomonas vaginalis in dry vaginal swabs. J Microbiol Methods. 2016;127:102-4.
19. Hoffman S, O'Sullivan LF, Harrison A, Dolezal C, Monroe-Wise A. HIV risk behaviors and the context of sexual coercion in young adults' sexual interactions: results from a diary study in rural South Africa. Sex Transm Dis. 2006;33(1):52-8.
20. Naidoo S, Wand H. Prevalence and incidence of Trichomonas vaginalis infections in women participating in a clinical trial in Durban, South Africa. Sex Transm Infect. 2013;89(6):519-22.
21. Bax CJ, Quint KD, Peters RP, Ouburg S, Oostvogel PM, Mutsaers JA, et al. Analyses of multiple-site and concurrent Chlamydia trachomatis serovar infections, and serovar tissue tropism for urogenital versus rectal specimens in male and female patients. Sex Transm Infect. 2011;87(6):503-7.
22. Verweij SP, Ouburg S, de Vries H, Morre SA, van Ginkel CJ, Bos H, et al. The first case record of a female patient with bubonic lymphogranuloma venereum (LGV), serovariant L2b. Sex Transm Infect. 2012;88(5):346-7.
23. Joffe GP, Foxman B, Schmidt AJ, Farris KB, Carter RJ, Neumann S, et al. Multiple partners and partner choice as risk factors for sexually transmitted disease among female college students. Sex Transm Dis. 1992;19(5):272-8.
24. Miller M, Liao Y, Gomez AM, Gaydos CA, D'Mellow D. Factors associated with the prevalence and incidence of Trichomonas vaginalis infection among African American women in New York city who use drugs. J Infect Dis. 2008;197(4):503-9.
25. Ginocchio CC, Chapin K, Smith JS, Aslanzadeh J, Snook J, Hill CS, et al. Prevalence of Trichomonas vaginalis and coinfection with Chlamydia trachomatis and Neisseria gonorrhoeae in the United States as determined by the Aptima Trichomonas vaginalis nucleic acid amplification assay. J Clin Microbiol. 2012;50(8):2601-8.
26. Munson E, Kramme T, Napierala M, Munson KL, Miller C, Hryciuk JE. Female epidemiology of transcription-mediated amplification-based Trichomonas vaginalis detection in a metropolitan setting with a high prevalence of sexually transmitted infection. J Clin Microbiol. 2012;50(12):3927-31.
27. Kwena Z, Sharma A, Wamae N, Muga C, Bukusi E. Provider characteristics among staff providing care to sexually transmitted infection self-medicating patients in retail pharmacies in Kibera slum, Nairobi, Kenya. Sex Transm Dis. 2008;35(5):480-3.
28. Sorvillo F, Smith L, Kerndt P, Ash L. Trichomonas vaginalis, HIV, and African-Americans. Emerg Infect Dis. 2001;7(6):927-32.
29. Chun HM, Carpenter RJ, Macalino GE, Crum-Cianflone NF. The Role of Sexually Transmitted Infections in HIV-1 Progression: A Comprehensive Review of the Literature. J Sex Transm Dis. 2013;2013:176459.
30. Fastring DR, Amedee A, Gatski M, Clark RA, Mena LA, Levison J, et al. Co-occurrence of Trichomonas vaginalis and bacterial vaginosis and vaginal shedding of HIV-1 RNA. Sex Transm Dis. 2014;41(3):173-9.
31. Fowke JH, Han X, Alderete JF, Moses KA, Signorello LB, Blot WJ. A prospective study of Trichomonas vaginalis and prostate cancer risk among African American men. BMC Res Notes. 2016;9:224.
32. Brotman RM. Vaginal microbiome and sexually transmitted infections: an epidemiologic perspective. J Clin Invest. 2011;121(12):4610-7.
33. Brotman RM, Klebanoff MA, Nansel TR, Yu KF, Andrews WW, Zhang J, et al. Bacterial vaginosis assessed by gram stain and diminished colonization resistance to incident gonococcal, chlamydial, and trichomonal genital infection. J Infect Dis. 2010;202(12):1907-15.

For peer review only

34. Neinstein LS, Goldenring J, Carpenter S. Nonsexual transmission of sexually transmitted diseases: an infrequent occurrence. Pediatrics. 1984;74(1):67-76.
35. Crucitti T, Jespers V, Mulenga C, Khondowe S, Vandepitte J, Buve A. Non-sexual transmission of Trichomonas vaginalis in adolescent girls attending school in Ndola, Zambia. PLoS One. 2011;6(1):e16310.
36. Nagarajan K, S G, Ramakrishnan L, Mainkar M, Ramesh S, Ganju D, et al. Self-Report of STI Symptoms, Inconsistent Condom use and Condom Nonuse are Poor Predictors of STI Prevalence among Men who have Sex with Men. Journal of AIDS & Clinical Research. 2013.
37. van der Eem L, Dubbink JH, Struthers HE, McIntyre JA, Ouburg S, Morre SA, et al. Evaluation of syndromic management guidelines for treatment of sexually transmitted infections in South African women. Trop Med Int Health. 2016;21(9):1138-46.
38. Rank RG, Yeruva L. Hidden in plain sight: chlamydial gastrointestinal infection and its relevance to persistence in human genital infection. Infect Immun. 2014;82(4):1362-71.
39. Francis SC, Kent CK, Klausner JD, Rauch L, Kohn R, Hardick A, et al. Prevalence of rectal Trichomonas vaginalis and Mycoplasma genitalium in male patients at the San Francisco STD clinic, 2005-2006. Sex Transm Dis. 2008;35(9):797-800.
40. Crucitti T, Jespers V, Mulenga C, Khondowe S, Vandepitte J, Buve A. Trichomonas vaginalis is highly prevalent in adolescent girls, pregnant women, and commercial sex workers in Ndola, Zambia. Sex Transm Dis. 2010;37(4):223-7.
41. Cosentino LA, Campbell T, Jett A, Macio I, Zamborsky T, Cranston RD, et al. Use of nucleic acid amplification testing for diagnosis of anorectal sexually transmitted infections. J Clin Microbiol. 2012;50(6):2005-8.
42. Jalal H, Verlander NQ, Kumar N, Bentley N, Carne C, Sonnex C. Genital chlamydial infection: association between clinical features, organism genotype and load. J Med Microbiol. 2011;60(Pt 7):881-8.
43. Gomes JP, Borrego MJ, Atik B, Santo I, Azevedo J, Brito de SA, et al. Correlating Chlamydia trachomatis infectious load with urogenital ecological success and disease pathogenesis. Microbes Infect. 2006;8(1):16-26.

For peer review only

Prevalence of Trichomonas vaginalis infection and protozoan load in South African women: a cross-sectional study

Journal:	*BMJ Open*
Manuscript ID	bmjopen-2017-016959.R3
Article Type:	Research
Date Submitted by the Author:	06-Sep-2017

Complete List of Authors:	de Waaij, Dewi; VU University Medical Centre, Laboratory of Immunogenetics, Dept. Medical Microbiology and Infection Control<br>Dubbink, Jan Henk; Anova Health Institute; University of Maastricht, Institute for Public Health Genomics, Faculty of Health, Medicine and LIfe Sciences<br>Ouburg, Sander; VU University Medical Centre, Laboratory of Immunogenetics, Dept. Medical Microbiology and Infection Control<br>Peters, Remco; Anova Health Institute, ; Maastricht University Medical Centre, Medical Microbiology<br>Morre, S; VU University medical center, Pathology, Laboratory of Immunogenetics

**Primary Subject Heading**:	Infectious diseases
Secondary Subject Heading:	Epidemiology, Infectious diseases, Sexual health
Keywords:	EPIDEMIOLOGY, INFECTIOUS DISEASES, Molecular diagnostics < INFECTIOUS DISEASES

SCHOLARONE™ Manuscripts

For peer review only

Prevalence of *Trichomonas vaginalis* infection and protozoan load in South African women: a cross-sectional study

Dewi J. de Waaij[1,2] , Jan Henk Dubbink[1,2], Sander Ouburg[1*], Remco P.H. Peters[3,4], Servaas A. Morré[1,2]

1 VU University Medical Centre, Department of Medical Microbiology & Infection Control, Laboratory of Immunogenetics, Amsterdam, The Netherlands.

2 Institute for Public Health Genomics (IPHG), Department of Genetics and Cell Biology, Research School GROW (School for Oncology & Developmental Biology), Faculty of Health, Medicine & Life Sciences, University of Maastricht, Maastricht, The Netherlands.

3 Anova Health Institute, Johannesburg and Tzaneen, South Africa.

4 Department of Medical Microbiology, University of Maastricht, Maastricht, The Netherlands.

* Corresponding author

dr. S. Ouburg, VU University Medical Center, Laboratory of Immunogenetics, dept. Medical Microbiology and Infection Control, De Boelelaan 1108, 1081HZ Amsterdam, the Netherlands. Tel.: +31 (0)20 444 8417, Fax.: +31 (0)20 444 8418. E-mail: s.ouburg@vumc.nl

Keywords: **Trichomonas vaginalis, risk factors, protozoan load, microbiological characteristics, rural South Africa**

Word count: 2841

Journal: BMJ open

For peer review only

ABSTRACT

Objectives. *Trichomonas vaginalis* is thought to be the most common non-viral sexually transmitted infection (STI) worldwide. We investigated the prevalence, risk factors and protozoan load of *Trichomonas vaginalis* (*T. vaginalis* load) infection in South African women.

Methods. A cross-sectional study of 604 women was conducted at 25 primary healthcare facilities in rural South Africa (Mopani district). *T. vaginalis* DNA was detected in vaginal and rectal swabs. In a uni- and multivariate analyses the *T. vaginalis* infection was investigated in relation to demographic characteristics, medical history, and behavioural factors. The *T. vaginalis* load was determined as the logarithm of DNA copies per microliter sample solution.

Results. Collected vaginal and rectal swabs were tested for *T. vaginalis* DNA. Prevalence of vaginal *T. vaginalis* was 20% (95% CI 17.0-23.4) and rectal 1.2% (95% CI 0.6-2.4). Most women (66%) with a vaginal infection were asymptomatic. Factors associated with *T. vaginalis* infection were a relationship status of single (OR, 2.4; 95% CI, 1.5-4.0; $P < 0.001$) and HIV positive-infection (OR, 1.6; 95% CI, 1.0-2.6; $P = 0.041$). Women with vaginal *T. vaginalis* infection were more likely to have concurrent *C. trachomatis* rectal infection than those without vaginal infection (12% vs. 3%; p<0.001, OR: 4.1). A higher median *T. vaginalis* load was observed among women with observed vaginal discharge compared to those without vaginal discharge ($P = 0.025$).

Conclusions. Vaginal trichomoniasis is highly prevalent in rural South Africa, especially among single women and those with HIV infection, and often presents without symptoms.

Strengths and limitations of this study

- This is the first study about the epidemiology and microbiological characteristics of *T. vaginalis* in rural South Africa
- The study has a high sample size
- The number of rectal *T. vaginalis* infections is relatively low
- A selection bias is possible based on the geographical inclusion

Data sharing: All data of this study have been described in the paper. No additional data for this study are available.

For peer review only

68 **Author contributions:**

69 DJdW: Laboratory analyses, statistical analyses, writing of the manuscript

70 JHD: Sample collection, writing of the manuscript

71 SO: Statistical analyses, reviewing of the manuscript

72 RPHP: Sample collection, reviewing of the manuscript, study supervision

73 SAM: reviewing of the manuscript, study supervision.

For peer review only

INTRODUCTION

Trichomonas vaginalis is a protozoan parasite that causes trichomoniasis and is mostly sexually transmitted. It is the most common non-viral sexually transmitted infection (STI) worldwide (1). The infection is asymptomatic in 85% of the women and in 77% of the men(1). The WHO describes that *T. vaginalis* in the African region occurs ten times as often in women than in men(2). Vaginal *T. vaginalis* infections in the entire African region is estimated to be 42.8 million(2). If the infection is symptomatic, women may report a change in vaginal discharge, intermenstrual bleeding, or vaginal blood loss during or after sexual contact. Other symptoms that can occur are vaginal itching, dysuria, and abdominal pain. Also upper reproductive tract disease syndromes can occur, including pelvic inflammatory disease. Women with a *T. vaginalis* infection have 4.7-fold increase in the risk of PID(3-5) and tubal pathology(6). Women infected with *T. vaginalis* also have a 1.3-fold increase in the risk of preterm labour(5).

In women, *T. vaginalis* infection can persist for months and, if left untreated, it can increase the risk for HIV acquisition if exposed(6, 7). *T. vaginalis* could lead to an increase in the vaginal HIV load and thus potentially increase the risk of HIV transmission to a sexual partner(8). In addition, an association between *T. vaginalis* and *Chlamydia trachomatis* infection has been found(9). Having a concurrent chlamydial infection was a predictor of both prevalent and incident *T. vaginalis*.

Despite the estimated large burden of *T. vaginalis* infection in the African region, data on clinical presentation, demographic and behavioural factors associated with infection, and microbiological factors is relatively limited. However, in other continents, multiple studies have been done in the context of *T. vaginalis* infections(10-13). *T. vaginalis* prevalence has been reported for a few African countries and ranges from 6.5-40%(14). One of the explanations for the often high *T. vaginalis* prevalence is the lack of STI screening programmes and limited control measurements. The estimated burden of disease is significant but available data of symptomatology, co-infections and pathogen load are limited. *C. trachomatis* and *Neisseria gonorrhoeae* are other important causative micro-organisms for STI, showing an association with the pathogen load as compared to the clinical presentation and the development of long-term sequelae(15, 16).

In this study, we present the epidemiology of *T. vaginalis* infection including the *T. vaginalis* load, as measured by a validated nucleic acid amplification technique. We studied a high HIV prevalence setting in rural South Africa.

For peer review only

METHODS

Population and Design

This study was part of a cross-sectional study of 604 women in rural Mopani District, South Africa, as described previously(17). In brief, women aged 18-49 years old who reported sexual activity during the prior 6 months were recruited at 25 primary healthcare (PHC) facilities, regardless of the reason for visiting the facility that day. After informed consent, demographic characteristics, medical history and details of sexual behaviour were collected using a questionnaire. Healthcare worker collected vaginal and rectal swabs were obtained for molecular testing of *T. vaginalis*.

The Human Ethics Research Committee of the University of the Witwatersrand, South Africa, approved the study (Ref M110726).

Laboratory testing

Collected vaginal and rectal swabs were analysed at the laboratory of Medical Microbiology & Infection Control, VU University Medical Centre, Amsterdam, the Netherlands. High Pure PCR Template Preparation Kit (HPPTP) (Roche Diagnostics, Basel, Switzerland) was used for the extraction of bacterial DNA. Detection of *T. vaginalis* DNA was done with the Presto^{plus} assay (Microbiome Ltd., Amsterdam, the Netherlands) and the LightCycler II 480 (Roche Diagnostics) was used for the quantitative PCR for *C. trachomatis*, *N. gonorrhoeae* and *T. vaginalis*(18). The Presto^{plus} assay for *C. trachomatis, N. gonorrhoeae,* and *T. vaginalis* is currently for in house use only and it will be transformed into a Presto-TV (Goffin Molecular Technologies, The Netherlands) in 2017 in a 200 reaction format as CE-IVD certified kit. PCR positivity was determined using the Cp (crossing point) value: we used a threshold Cp of <38 for a sample to be classified as positive. Samples with Cp values of ≥38 were retested and if again ≥38 were excluded from further analyses (indeterminate samples).

Data analyses

The following risk factors were used in our analyses: age, being single, unemployment, current pregnancy, history of vaginal discharge syndrome (VDS), currently being pregnant, the use of hormonal contraceptives, administering intravaginal cleansing, visiting bars, alcohol use, concurrent partners, condom use during last sex act, partner >10 years older, experiencing coercion and/or force, and sex for money or for other benefits(17). Coercion reflects sex inequality in relationships which is associated with increased risk(19).

For peer review only - http://bmjopen.bmj.com/site/about/guidelines.xhtml

Data were double-entered in Epi Data (Epi Info^{TM} version 3.5.3) by two researchers independently, were compared for inconsistencies, cleaned and verified. The data were analysed using SPSS version 20.0 (SPSS Inc., Chicago, USA). Dichotomous data were compared using χ^2 test or Fisher's Exact test and continuous data using the Mann-Whitney *U* test. Odds ratios (ORs) with 95% confidence interval (CI) were calculated. Vaginal *T. vaginalis* infection was defined symptomatic when either abnormal vaginal discharge during physical examination, intermenstrual bleeding, or vaginal blood loss during or after sexual intercourse was reported. Univariate analysis was performed to examine possible associations of demographic variables and clinical and behavioural factors in relation to *T. vaginalis*. Multivariate analysis was conducted using a backward selection procedure and likelihood ratio tests. As a proxy for the *T. vaginalis* load we used the median Cp value, which was calculated for each risk factor and compared between infected versus non-infected women.

For peer review only

RESULTS

Demographics of study population

A quarter of all women (n= 154; 25.5%) had an age 18-24, over half of the women were single (334; (55.3%), 26 women reported anal intercourse (4.3%), a high proportion was unemployed, and almost 30% reported to be infected with HIV (177; 29.3%). For more details see **Table 1**.

Prevalence of *T. vaginalis* infection

Of the 604 vaginal samples, one sample was excluded based on lack of biological material and 28 gave an indeterminate result (2 times Cp values of ≥38); these were excluded resulting in 575 samples for analysis. For vaginal infection, 113/575 (20%) samples tested positive.

Of the 604 rectal samples, nine samples gave an indeterminate result (2 times Cp values of ≥38) and were excluded resulting in 595 samples. For rectal infection, 7/595 (1.2%) samples tested positive. Four patients were infected *T. vaginalis* at both the rectal and vaginal site.

Clinical manifestation of infection

Symptoms of vaginal infection were reported by 196 (34%) of the women. Of these, 38 had *T. vaginalis* infection meaning 38/113 (34%) of the women with TV reported symptoms. Of the 462 women without *T. vaginalis,* 158 reported symptoms of infection (34%). None of the individual symptoms (vaginal discharge, intermenstrual bleeding and vaginal blood loss related to sexual contact) was associated with *T. vaginalis* infection. For rectal infection (n=7) the numbers were low and no rectal symptomatology was scored.

Factors associated with *Trichomonas vaginalis* infection

In univariate analyses we observed significant associations for single status, being HIV positive, the use of hormonal contraceptives, and visiting bars (**Table 1**). In multivariate analysis (**Table 1**), having a relationship status that is single was significantly associated with a vaginal *T. vaginalis* infection (adjusted OR [aOR], 2.4 [95% CI, 1.5-4.0, p <0.001]) and presence of HIV infection was significantly associated (aOR, 1.6; [95% CI 1.0-2.6; p = 0.041]) *T. vaginalis* infection. Within women with a current vaginal *T. vaginalis* infection, a rectal *C. trachomatis* infection is significantly more present (P<0.001, OR: 4.1).

For peer review only

Table 1. Univariate and Multivariate Analysis of Factors associated with Vaginal *Trichomonas vaginalis* Infection in South African Women

			Univariate		Multivariate	
Factor	n	%	OR (95% CI)	*P*	OR (95% CI)	*P*
Age 18-24	154	25.5	0.9 (0.6-1.6)	0.95	-	NS
Single	334	55.3	2.4 (1.5-3.9)	**<0.001**	2.4 (1.5-4.0)	**<0.001**
Unemployed	424	70.2	1.5 (0.9-2.4)	0.135	-	NS
HIV infected	177	29.3	1.6 (1.0-2.4)	**0.040**	1.6 (1.0-2.6)	**0.041**
History of VDS*	196	32.4	1.0 (0.7-1.6)	0.878	-	NS
Currently pregnant	94	15.9	1.6 (1.0-2.7)	0.067	-	NS
Hormonal contraceptives	230	33.6	0.6 (0.4-0.9)	**0.016**	-	NS
Intravaginal cleansing	132	21.9	1.0 (0.6-1.6)	0.841	-	NS
Visits bars	76	12.6	1.8 (1.0-3.1)	**0.027**	1.7 (0.96-3.1)	NS
Alcohol use	12	2	1.4 (0.4-5.1)	0.640	-	NS
Concurrent partners	85	14	1.1 (0.7-1.6)	0.799	-	NS
Condom use last act	207	34.3	1.2 (0.8-1.8)	0.487	-	NS
Partner >10 y older	202	33.4	1.2 (0.8-1.9)	0.335	-	NS
Experienced coercion**	169	28	0.7 (0.4-1.1)	0.142	0.6 (0.4-1.0)	0.058
Experienced force	38	6.3	1.3 (0.6-2.8)	0.541	-	NS
Sex for money or other benefits	10	1.7	1.3 (0.5-3.3)	0.579	-	NS

**VDS contains reported change in vaginal discharge, intermenstrual bleeding, or vaginal blood loss during or after sexual contact*

***Coercion reflects sex inequality in relationships, associated with increased risk*(17)

For peer review only

Determinants of *T. vaginalis* load

The median Cp value for vaginal *T. vaginalis* load of infection was 28.0 (range 17.0 – 37.9). We did not observe any significant associations between the *T. vaginalis* load and demographic factors (**Table 2**). Also no association was found between the *T. vaginalis* load and symptoms in general. However, in the group of women with *T. vaginalis* infection who had vaginal discharge observed during examination, a significantly higher load was observed as compared to those without vaginal discharge (*P* = 0.025).

Table 2. *T. vaginalis* load in association with risk factors

Risk factor		Median Cp	*P*
Age 18-24	18-24	28	0.747
	>24	27.7	
Single	Yes	27.3	0.971
	No	28	
Unemployed	Yes	27.7	0.810
	No	29.7	
HIV infected	Yes	27.5	0.986
	No	27.8	
History of VDS	Yes	28	0.954
	No	27.6	
Currently pregnant	Yes	28	0.794
	No	27.7	
Hormonal contraceptives	Yes	27.6	0.464
	No	28	
Intravaginal cleansing	Yes	28.5	0.433
	No	27.7	
Visits bars	Yes	25.3	0.310
	No	28	
Alcohol use	Yes	27.9	0.613
	No	30.2	
Concurrent partners	Yes	27	0.335
	No	28	
Condom use last sex act	Yes	27	0.339
	No	28	
Experiences force	Yes	29	0.603
	No	27.9	

For peer review only

DISCUSSION

In this study, we describe the prevalence, risk factors, and the load of *T. vaginalis* infection. This is one of a few studies to report these aspects for *T. vaginalis* in a cohort from South Africa. We observed a vaginal *T. vaginalis* prevalence of 20% (95% CI 17.0-23.4) and a rectal prevalence of 1.2% (95% CI 0.6-2.4).

In multivariate analysis two risk factors for vaginal *T. vaginalis* infection were identified: having a relationship status that is single and the presence of HIV infection. In addition, within women with vaginal *T. vaginalis* infection, a rectal *C. trachomatis* infection is significantly more present. Finally, a high *T. vaginalis* load appears to be associated with vaginal discharge observed during examination.

The prevalence of vaginal *T. vaginalis* infection in our study corresponds with the range reported by other studies in Africa (3.2%-40%)(14, 20). Rectal *T. vaginalis* infections are rarely described. One study from San Francisco, USA, describes a rectal *T. vaginalis* prevalence of 0.6%, based on 'men who have sex with men' (MSM) who reported receptive anal sex within six months prior to their clinic visit(21, 22).

We show two demographic factors that may contribute to the high vaginal *T. vaginalis* prevalence in our study. First, we observed that a *T. vaginalis* infection is associated with a single relationship status. Joffe *et al.* described that a single marital status is often associated with having more than one sexual partner and thereby an increased risk for STI in general(23). To be more specific, it has been demonstrated that women having more than two concurrent male sexual partners are more likely to have a *T. vaginalis* infection(24). Although we were not able to confirm this, we hypothesize that a single marital status is associated with having more than one concurrent sexual partner.

It has been suggested that *T. vaginalis* infection are age-tied(25, 26). Women who were older than 20 years of age, were more likely to be infected with *T. vaginalis*(27). In our study we did not observe an association between age an T. vaginalis infections.

We confirm an association of positive HIV status with presence of *T. vaginalis* infection, which has been reported by several other studies(4, 8, 28-31). *T. vaginalis* infection was present in almost a quarter of HIV-infected women in our study. This is in line with the previously described higher trichomonas prevalence in high HIV prevalence cities compared to low HIV prevalence cities(14, 20). The biological link between HIV and TV is yet unclear, however bacterial vaginosis, is described to be an independent risk factor for acquisition of STI, and acquisition and transmission of HIV(32). Although we did not include bacterial vaginosis, it is described to be associated with a 1.5-2-fold increased risk for incident trichomonal infection(33). This could suggest an association between HIV and *T. vaginalis* where an asymptomatic *T. vaginalis* infection could increase the risk of HIV infection.

For peer review only

It has been described that non-sexual transmission of *T. vaginalis* occurs in girls who did not have sexual intercourse(34, 35). A striking prevalence found by a study among women from Ndola, Zambia, who denied ever had sexual intercourse was 43%(14). In those cases, *T. vaginalis* would be transmitted by wet clothes and water, since some studies suggest that *T. vaginalis* can survive up to days in warm mineral water and also for hours in urine(34). *T. vaginalis* is described to have some kind of robustness, which may explain its different transmission ways, compared to other STI. This may be an explanation of many hypotheses for the high prevalence, however, they need to be further explored.

Twenty-six percent of all women reported symptoms, without being diagnosed with a *T. vaginalis* infection. Although 20% of these women were diagnosed with another vaginal STI than *T. vaginalis*, symptoms are evidently a poor predictor for a *T. vaginalis* infection. We did not find a significant difference in symptomatology and *T. vaginalis* infection status. More knowledge about *T. vaginalis* disease would provide valuable information for potential targeted treatment approaches versus syndromic management.

In general, self-reported symptoms are not reliable in predicting an STI, like *T. vaginalis*(36, 37). However, 31 (16%) of the women vaginally infected with *T. vaginalis* were co-infected with either *C. trachomatis* or *N. gonorrhoeae* (data not shown). Symptoms of these frequent occurring STI may show comparable symptoms and should be taken into consideration. Although other studies revealed significant rates for co-infection between *T. vaginalis* and other STIs [12804936], in the current study we did not observe an increased risk for co-infection with *C. trachomatis* and/or *N. gonorrhoeae* in *T. vaginalis* infected patients (data not shown). However, in a previous study from our group we describe an increased risk of obtaining a *T. vaginalis* coinfection when infected with either *C. trachomatis* or *N. gonorrhoeae*. Concurrent STIs may be due to common risk factors for these infections, such as sexual activity, contraceptive use, and new partners.

This is one of a few studies to report aspects of rectal *T. vaginalis* in a cohort from South Africa. We observed that rectal *C. trachomatis* infections are significant more present in combination with a vaginal *T. vaginalis* infection. This may be due to several factors. First, sexual behaviour including anal sex is an important factor that may play a role in the presence of co-infections. Second, there is the ability to get re-infected via the gastrointestinal tract by auto inoculation, after being cured of the vaginal infection for Chlamydia, but it is unclear if this route of infection is also present for *T. vaginalis* infections and thus if the rectal site could be the reservoir for *T. vaginalis* infection(38). These potential transmission routes may result from vaginal wiping techniques, poor personal hygiene, using fingers during sex or for cleansing afterward, and use of sex toys(17). The most likely explanation is that the rectal samples may be contaminated by cervicovaginal fluid due to the close

For peer review only

anatomical proximity(39, 40) and that *T. vaginalis* positive rectal samples in women were contaminated with *T. vaginalis* positive vaginal fluid of the perianal surface(41).

It is conceivable that microorganism load may affect the clinical course of infection. It has been shown in studies of another STI, *C. trachomatis*, that there is an association between the bacterial load and genital manifestations of infection(42, 43). A higher *C. trachomatis* bacterial load is associated with the presence of at least two specific symptoms (vaginal discharge, irregular bleeding, or pelvic or abdominal pain). The impact of *T. vaginalis* load has not yet been described. We hypothesized that *T. vaginalis* load may affect the clinical course of the infection and compared several factors with the load of *T. vaginalis*. We did not find any significant association between the load and reported symptoms of a *T. vaginalis* infection. However, within women with observed vaginal discharge during examination, a significantly higher load was found. The load determination was performed using Cp values of the *T. vaginalis* PCR. Another technique to determine the average load per human is to incorporate the number of human cells. Our results prove that there is an association between *T. vaginalis* load and discharge. However, the literature on *T. vaginalis* load is scarce, and the effect of the load has to be further explored.

Although this is a unique study about the epidemiology and microbiological characteristics of *T. vaginalis* in rural South Africa with a relatively good sample size, a limitation of our study is that the number of rectal *T. vaginalis* infections is rather small for further analyses. As a consequence, this could have played a role in our statistical analyses. In addition, there can be a selection bias based on for instance the geographical inclusion, and self-reported symptoms can be more subjective than clinician based symptomatology. We cannot exclude potential cross-contamination between the cervico-vaginal and ano-rectal sites. The results of this may be generalised to similar populations however these results might not be extended to other populations due to the aforementioned potential geographical and reporting biases.

In conclusion, *T. vaginalis* infections are highly prevalent in the rural South African population investigated. The asymptomatic nature, potential for non-sexual transmission especially in socio-economic poor settings, and the syndromic management approach to STI control in this region are potential factors contributing to the prevalence. We observed a low rectal prevalence of which might be contamination with *T. vaginalis* positive vaginal fluid of the perianal surface. In multivariate analyses being single or HIV positive were identified as risk factors for vaginal *T. vaginalis* infection. Finally, a high *T. vaginalis* load seems associated with vaginal discharge observed during examination.

For peer review only

Funding

This work was supported by the Dutch Society for Tropical Medicine (NVTG), the Netherlands. The Anova Health Institute is supported by the US President's Emergency Plan for AIDS Relief program via the US Agency for International Development under Cooperative Agreement No. AID-674-A-12-00015. The views expressed in this manuscript do not necessarily reflect those of the President's Emergency Plan for AIDS Relief or the US Agency for International Development.

CONFLICT OF INTEREST

S.A.M. is a fulltime employee of the VU University Medical Center Amsterdam, and has been involved in the technical development of the Presto^{plus} CT-NG-TV assay (Microbiome, The Netherlands) via Microbiome Ltd. (S.A.M. is co-founder and co-director), a spin-off company of the VU University Medical Center, Amsterdam, the Netherlands.

None of the other authors report a potential conflict of interest.

For peer review only

1. Kissinger P. Epidemiology and treatment of trichomoniasis. Curr Infect Dis Rep. 2015;17(6):484.
2. World Health Organization DoRHaR. Global incidence and prevalence of selected curable sexually transmitted infections - 2008. 2012 2012. Report No.
3. Moodley P, Wilkinson D, Connolly C, Moodley J, Sturm AW. Trichomonas vaginalis is associated with pelvic inflammatory disease in women infected with human immunodeficiency virus. Clin Infect Dis. 2002;34(4):519-22.
4. Kissinger P, Adamski A. Trichomoniasis and HIV interactions: a review. Sex Transm Infect. 2013;89(6):426-33.
5. Cotch MF, Pastorek JG, Nugent RP, Hillier SL, Gibbs RS, Martin DH, et al. Trichomonas vaginalis associated with low birth weight and preterm delivery. The Vaginal Infections and Prematurity Study Group. Sex Transm Dis. 1997;24(6):353-60.
6. Soper D. Trichomoniasis: under control or undercontrolled? Am J Obstet Gynecol. 2004;190(1):281-90.
7. Van Der Pol B, Williams JA, Orr DP, Batteiger BE, Fortenberry JD. Prevalence, incidence, natural history, and response to treatment of Trichomonas vaginalis infection among adolescent women. J Infect Dis. 2005;192(12):2039-44.
8. Van Der Pol B. Trichomonas vaginalis infection: the most prevalent nonviral sexually transmitted infection receives the least public health attention. Clin Infect Dis. 2007;44(1):23-5.
9. Helms DJ, Mosure DJ, Metcalf CA, Douglas JM, Jr., Malotte CK, Paul SM, et al. Risk factors for prevalent and incident Trichomonas vaginalis among women attending three sexually transmitted disease clinics. Sex Transm Dis. 2008;35(5):484-8.
10. Schwebke JR, Hobbs MM, Taylor SN, Sena AC, Catania MG, Weinbaum BS, et al. Molecular testing for Trichomonas vaginalis in women: results from a prospective U.S. clinical trial. J Clin Microbiol. 2011;49(12):4106-11.
11. van Der Schee C, van Belkum A, Zwijgers L, van Der Brugge E, O'Neill E L, Luijendijk A, et al. Improved diagnosis of Trichomonas vaginalis infection by PCR using vaginal swabs and urine specimens compared to diagnosis by wet mount microscopy, culture, and fluorescent staining. J Clin Microbiol. 1999;37(12):4127-30.
12. Sena AC, Miller WC, Hobbs MM, Schwebke JR, Leone PA, Swygard H, et al. Trichomonas vaginalis infection in male sexual partners: implications for diagnosis, treatment, and prevention. Clin Infect Dis. 2007;44(1):13-22.
13. Vatanshenassan M, Rezaie S, Mohebali M, Niromand N, Kazemi B, Babaei Z, et al. Trichomonas vaginalis: investigation of a novel diagnostic method in urine samples using cysteine proteinase 4 gene and PCR technique. Exp Parasitol. 2010;126(2):187-90.
14. Buve A, Weiss HA, Laga M, Van DE, Musonda R, Zekeng L, et al. The epidemiology of trichomoniasis in women in four African cities. AIDS. 2001;15 Suppl 4:S89-S96.
15. Michel CE, Sonnex C, Carne CA, White JA, Magbanua JP, Nadala EC, Jr., et al. Chlamydia trachomatis load at matched anatomic sites: implications for screening strategies. J Clin Microbiol. 2007;45(5):1395-402.
16. Bissessor M, Tabrizi SN, Fairley CK, Danielewski J, Whitton B, Bird S, et al. Differing Neisseria gonorrhoeae bacterial loads in the pharynx and rectum in men who have sex with men: implications for gonococcal detection, transmission, and control. J Clin Microbiol. 2011;49(12):4304-6.
17. Peters RP, Dubbink JH, van der Eem L, Verweij SP, Bos ML, Ouburg S, et al. Cross-sectional study of genital, rectal, and pharyngeal Chlamydia and gonorrhea in women in rural South Africa. Sex Transm Dis. 2014;41(9):564-9.

For peer review only

18. de Waaij DJ, Ouburg S, Dubbink JH, Peters RP, Morre SA. Evaluation of Presto(plus) assay and LightMix kit Trichomonas vaginalis assay for detection of Trichomonas vaginalis in dry vaginal swabs. J Microbiol Methods. 2016;127:102-4.
19. Hoffman S, O'Sullivan LF, Harrison A, Dolezal C, Monroe-Wise A. HIV risk behaviors and the context of sexual coercion in young adults' sexual interactions: results from a diary study in rural South Africa. Sex Transm Dis. 2006;33(1):52-8.
20. Naidoo S, Wand H. Prevalence and incidence of Trichomonas vaginalis infections in women participating in a clinical trial in Durban, South Africa. Sex Transm Infect. 2013;89(6):519-22.
21. Bax CJ, Quint KD, Peters RP, Ouburg S, Oostvogel PM, Mutsaers JA, et al. Analyses of multiple-site and concurrent Chlamydia trachomatis serovar infections, and serovar tissue tropism for urogenital versus rectal specimens in male and female patients. Sex Transm Infect. 2011;87(6):503-7.
22. Verweij SP, Ouburg S, de Vries H, Morre SA, van Ginkel CJ, Bos H, et al. The first case record of a female patient with bubonic lymphogranuloma venereum (LGV), serovariant L2b. Sex Transm Infect. 2012;88(5):346-7.
23. Joffe GP, Foxman B, Schmidt AJ, Farris KB, Carter RJ, Neumann S, et al. Multiple partners and partner choice as risk factors for sexually transmitted disease among female college students. Sex Transm Dis. 1992;19(5):272-8.
24. Miller M, Liao Y, Gomez AM, Gaydos CA, D'Mellow D. Factors associated with the prevalence and incidence of Trichomonas vaginalis infection among African American women in New York city who use drugs. J Infect Dis. 2008;197(4):503-9.
25. Ginocchio CC, Chapin K, Smith JS, Aslanzadeh J, Snook J, Hill CS, et al. Prevalence of Trichomonas vaginalis and coinfection with Chlamydia trachomatis and Neisseria gonorrhoeae in the United States as determined by the Aptima Trichomonas vaginalis nucleic acid amplification assay. J Clin Microbiol. 2012;50(8):2601-8.
26. Munson E, Kramme T, Napierala M, Munson KL, Miller C, Hryciuk JE. Female epidemiology of transcription-mediated amplification-based Trichomonas vaginalis detection in a metropolitan setting with a high prevalence of sexually transmitted infection. J Clin Microbiol. 2012;50(12):3927-31.
27. Kwena Z, Sharma A, Wamae N, Muga C, Bukusi E. Provider characteristics among staff providing care to sexually transmitted infection self-medicating patients in retail pharmacies in Kibera slum, Nairobi, Kenya. Sex Transm Dis. 2008;35(5):480-3.
28. Sorvillo F, Smith L, Kerndt P, Ash L. Trichomonas vaginalis, HIV, and African-Americans. Emerg Infect Dis. 2001;7(6):927-32.
29. Chun HM, Carpenter RJ, Macalino GE, Crum-Cianflone NF. The Role of Sexually Transmitted Infections in HIV-1 Progression: A Comprehensive Review of the Literature. J Sex Transm Dis. 2013;2013:176459.
30. Fastring DR, Amedee A, Gatski M, Clark RA, Mena LA, Levison J, et al. Co-occurrence of Trichomonas vaginalis and bacterial vaginosis and vaginal shedding of HIV-1 RNA. Sex Transm Dis. 2014;41(3):173-9.
31. Fowke JH, Han X, Alderete JF, Moses KA, Signorello LB, Blot WJ. A prospective study of Trichomonas vaginalis and prostate cancer risk among African American men. BMC Res Notes. 2016;9:224.
32. Brotman RM. Vaginal microbiome and sexually transmitted infections: an epidemiologic perspective. J Clin Invest. 2011;121(12):4610-7.
33. Brotman RM, Klebanoff MA, Nansel TR, Yu KF, Andrews WW, Zhang J, et al. Bacterial vaginosis assessed by gram stain and diminished colonization resistance to incident gonococcal, chlamydial, and trichomonal genital infection. J Infect Dis. 2010;202(12):1907-15.

For peer review only

34. Neinstein LS, Goldenring J, Carpenter S. Nonsexual transmission of sexually transmitted diseases: an infrequent occurrence. Pediatrics. 1984;74(1):67-76.
35. Crucitti T, Jespers V, Mulenga C, Khondowe S, Vandepitte J, Buve A. Non-sexual transmission of Trichomonas vaginalis in adolescent girls attending school in Ndola, Zambia. PLoS One. 2011;6(1):e16310.
36. Nagarajan K, S G, Ramakrishnan L, Mainkar M, Ramesh S, Ganju D, et al. Self-Report of STI Symptoms, Inconsistent Condom use and Condom Nonuse are Poor Predictors of STI Prevalence among Men who have Sex with Men. Journal of AIDS & Clinical Research. 2013.
37. van der Eem L, Dubbink JH, Struthers HE, McIntyre JA, Ouburg S, Morre SA, et al. Evaluation of syndromic management guidelines for treatment of sexually transmitted infections in South African women. Trop Med Int Health. 2016;21(9):1138-46.
38. Rank RG, Yeruva L. Hidden in plain sight: chlamydial gastrointestinal infection and its relevance to persistence in human genital infection. Infect Immun. 2014;82(4):1362-71.
39. Francis SC, Kent CK, Klausner JD, Rauch L, Kohn R, Hardick A, et al. Prevalence of rectal Trichomonas vaginalis and Mycoplasma genitalium in male patients at the San Francisco STD clinic, 2005-2006. Sex Transm Dis. 2008;35(9):797-800.
40. Crucitti T, Jespers V, Mulenga C, Khondowe S, Vandepitte J, Buve A. Trichomonas vaginalis is highly prevalent in adolescent girls, pregnant women, and commercial sex workers in Ndola, Zambia. Sex Transm Dis. 2010;37(4):223-7.
41. Cosentino LA, Campbell T, Jett A, Macio I, Zamborsky T, Cranston RD, et al. Use of nucleic acid amplification testing for diagnosis of anorectal sexually transmitted infections. J Clin Microbiol. 2012;50(6):2005-8.
42. Jalal H, Verlander NQ, Kumar N, Bentley N, Carne C, Sonnex C. Genital chlamydial infection: association between clinical features, organism genotype and load. J Med Microbiol. 2011;60(Pt 7):881-8.
43. Gomes JP, Borrego MJ, Atik B, Santo I, Azevedo J, Brito de SA, et al. Correlating Chlamydia trachomatis infectious load with urogenital ecological success and disease pathogenesis. Microbes Infect. 2006;8(1):16-26.

For peer review only

STROBE Statement—checklist of items that should be included in reports of observational studies

	Item No.	Recommendation	Page No.	Relevant text from manuscript
**Title and abstract**	1	(*a*) Indicate the study's design with a commonly used term in the title or the abstract	1	a cross-sectional study
		(*b*) Provide in the abstract an informative and balanced summary of what was done and what was found	2	Prevalence of vaginal T. vaginalis was 20% (95% CI 17.0-23.4) and rectal 1.2% (95% CI 0.6-2.4). Most women (66%) with a vaginal infection were asymptomatic. Factors associated with T. vaginalis infection were a relationship status of single (OR, 2.4; 95% CI, 1.5-4.0; P < 0.001) and HIV positive-infection (OR, 1.6; 95% CI, 1.0-2.6; P = 0.041). Women with vaginal T. vaginalis infection were more likely to have concurrent C. trachomatis rectal infection than those without vaginal infection (12% vs. 3%; p<0.001, OR: 4.1). A higher median T. vaginalis load was observed among women with observed vaginal discharge compared to those without vaginal discharge (P = 0.025).
**Introduction**				
Background/rationale	2	Explain the scientific background and rationale for the investigation being reported	4	*Trichomonas vaginalis* is a protozoan parasite that causes trichomoniasis and is mostly sexually transmitted. It is the most common non-viral sexually transmitted infection (STI) worldwide (1). The infection is asymptomatic in 85% of the women and in 77%

For peer review only - http://bmjopen.bmj.com/site/about/guidelines.xhtml

of the men(1). The WHO describes that *T. vaginalis* in the African region occurs ten times as often in women than in men(2). Vaginal *T. vaginalis* infections in the entire African region is estimated to be 42.8 million(2). If the infection is symptomatic, women may report a change in vaginal discharge, intermenstrual bleeding, or vaginal blood loss during or after sexual contact. Other symptoms that can occur are vaginal itching, dysuria, and abdominal pain. Also upper reproductive tract disease syndromes can occur, including pelvic inflammatory disease. Women with a *T. vaginalis* infection have 4.7-fold increase in the risk of PID(3-5) and tubal pathology(6). Women infected with *T. vaginalis* also have a 1.3-fold increase in the risk of preterm labour(5).

In women, *T. vaginalis* infection can persist for months and, if left untreated, it can increase the risk for HIV acquisition if exposed(6, 7). *T. vaginalis* could lead to an increase in the vaginal HIV load and thus potentially increase the risk of HIV transmission to a sexual partner(8). In addition, an association between *T. vaginalis* and *Chlamydia trachomatis* infection has been found(9). Having a concurrent chlamydial infection was a predictor of both

For peer review only - http://bmjopen.bmj.com/site/about/guidelines.xhtml

For peer review only

				prevalent and incident *T. vaginalis*. Despite the estimated large burden of *T. vaginalis* infection in the African region, data on clinical presentation, demographic and behavioural factors associated with infection, and microbiological factors is relatively limited. However, in other continents, multiple studies have been done in the context of *T. vaginalis* infections(10-13). *T. vaginalis* prevalence has been reported for a few African countries and ranges from 6.5-40%(14). One of the explanations for the often high *T. vaginalis* prevalence is the lack of STI screening programmes and limited control measurements. The estimated burden of disease is significant but available data of symptomatology, co-infections and pathogen load are limited. *C. trachomatis* and *Neisseria gonorrhoeae* are other important causative micro-organisms for STI, showing an association with the pathogen load as compared to the clinical presentation and the development of long-term sequelae(15, 16).
Objectives	3	State specific objectives, including any prespecified hypotheses	4	In this study, we present the epidemiology of T. vaginalis infection including the T. vaginalis load, as measured by a validated nucleic acid amplification technique. We

For peer review only - http://bmjopen.bmj.com/site/about/guidelines.xhtml

				studied a high HIV prevalence setting in rural South Africa.
**Methods**				
Study design	4	Present key elements of study design early in the paper	5	This study was part of a cross-sectional study of 604 women in rural Mopani District, South Africa, as described previously(17).
Setting	5	Describe the setting, locations, and relevant dates, including periods of recruitment, exposure, follow-up, and data collection	5	This study was part of a cross-sectional study of 604 women in rural Mopani District, South Africa, as described previously(17). In brief, women aged 18-49 years old who reported sexual activity during the prior 6 months were recruited at 25 primary healthcare (PHC) facilities, regardless of the reason for visiting the facility that day. After informed consent, demographic characteristics, medical history and details of sexual behaviour were collected using a questionnaire. Healthcare worker collected vaginal and rectal swabs were obtained for molecular testing of T. vaginalis.
Participants	6	(*a*) *Cohort study*—Give the eligibility criteria, and the sources and methods of selection of participants. Describe methods of follow-up<br>*Case-control study*—Give the eligibility criteria, and the sources and methods of case ascertainment and control selection. Give the rationale for the choice of cases and controls<br>*Cross-sectional study*—Give the eligibility criteria, and the sources and methods of selection of participants	5	women aged 18-49 years old who reported sexual activity during the prior 6 months were recruited at 25 primary healthcare (PHC) facilities, regardless of the reason for visiting the facility that day. After informed consent, demographic characteristics, medical history and details of sexual behaviour were collected using a questionnaire.

For peer review only - http://bmjopen.bmj.com/site/about/guidelines.xhtml

		(*b*) *Cohort study*—For matched studies, give matching criteria and number of exposed and unexposed<br>*Case-control study*—For matched studies, give matching criteria and the number of controls per case	5	
Variables	7	Clearly define all outcomes, exposures, predictors, potential confounders, and effect modifiers. Give diagnostic criteria, if applicable	5	PCR positivity was determined using the Cp (crossing point) value: we used a threshold Cp of <38 for a sample to be classified as positive. Samples with Cp values of ≥38 were retested and if again ≥38 were excluded from further analyses (indeterminate samples).<br><br>The following risk factors were used in our analyses: age, being single, unemployment, current pregnancy, history of vaginal discharge syndrome (VDS), currently being pregnant, the use of hormonal contraceptives, administering intravaginal cleansing, visiting bars, alcohol use, concurrent partners, condom use during last sex act, partner >10 years older, experiencing coercion and/or force, and sex for money or for other benefits(17). Coercion reflects sex inequality in relationships which is associated with increased risk(19).<br>Data were double-entered in Epi Data (Epi InfoTM version 3.5.3) by two researchers independently, were compared for inconsistencies, cleaned and verified. The data were analysed using SPSS version 20.0 (SPSS Inc., Chicago, USA).

For peer review only - http://bmjopen.bmj.com/site/about/guidelines.xhtml

				Dichotomous data were compared using $\chi 2$ test or Fisher's Exact test and continuous data using the Mann-Whitney U test. Odds ratios (ORs) with 95% confidence interval (CI) were calculated. Vaginal T. vaginalis infection was defined symptomatic when either abnormal vaginal discharge during physical examination, intermenstrual bleeding, or vaginal blood loss during or after sexual intercourse was reported. Univariate analysis was performed to examine possible associations of demographic variables and clinical and behavioural factors in relation to T. vaginalis. Multivariate analysis was conducted using a backward selection procedure and likelihood ratio tests. As a proxy for the T. vaginalis load we used the median Cp value, which was calculated for each risk factor and compared between infected versus non-infected women.
Data sources/ measurement	8*	For each variable of interest, give sources of data and details of methods of assessment (measurement). Describe comparability of assessment methods if there is more than one group	5	See 7
Bias	9	Describe any efforts to address potential sources of bias	5	See 7
Study size	10	Explain how the study size was arrived at	5	See 7

Continued on next page

For peer review only - http://bmjopen.bmj.com/site/about/guidelines.xhtml

Quantitative variables	11	Explain how quantitative variables were handled in the analyses. If applicable, describe which groupings were chosen and why	6	Dichotomous data were compared using χ2 test or Fisher's Exact test and continuous data using the Mann-Whitney U test. Odds ratios (ORs) with 95% confidence interval (CI) were calculated. . Univariate analysis was performed to examine possible associations of demographic variables and clinical and behavioural factors in relation to T. vaginalis. Multivariate analysis was conducted using a backward selection procedure and likelihood ratio tests. As a proxy for the T. vaginalis load we used the median Cp value, which was calculated for each risk factor and compared between infected versus non-infected women.
Statistical methods	12	(*a*) Describe all statistical methods, including those used to control for confounding	6	See 11
		(*b*) Describe any methods used to examine subgroups and interactions	6	See 11
		(*c*) Explain how missing data were addressed	7	See 13
		(*d*) *Cohort study*—If applicable, explain how loss to follow-up was addressed<br>*Case-control study*—If applicable, explain how matching of cases and controls was addressed<br>*Cross-sectional study*—If applicable, describe analytical methods taking account of sampling strategy	6	
		(*e*) Describe any sensitivity analyses		
**Results**				
Participants	13*	(a) Report numbers of individuals at each stage of study—eg numbers potentially eligible, examined for eligibility, confirmed eligible, included in the study, completing follow-up, and analysed	7	A quarter of all women (n= 154; 25.5%) had an age 18-24, over half of the women were single (334; (55.3%), 26 women reported anal intercourse (4.3%), a high proportion was unemployed, and almost 30% reported to be infected with HIV (177; 29.3%). For more details see Table 1.

For peer review only - http://bmjopen.bmj.com/site/about/guidelines.xhtml

Prevalence of T. vaginalis infection
Of the 604 vaginal samples, one sample was excluded based on lack of biological material and 28 gave an indeterminate result (2 times Cp values of ≥38); these were excluded resulting in 575 samples for analysis. For vaginal infection, 113/575 (20%) samples tested positive.
Of the 604 rectal samples, nine samples gave an indeterminate result (2 times Cp values of ≥38) and were excluded resulting in 595 samples. For rectal infection, 7/595 (1.2%) samples tested positive. Four patients were infected T. vaginalis at both the rectal and vaginal site.

Clinical manifestation of infection
Symptoms of vaginal infection were reported by 196 (34%) of the women. Of these, 38 had T. vaginalis infection meaning 38/113 (34%) of the women with TV reported symptoms. Of the 462 women without T. vaginalis, 158 reported symptoms of infection (34%). None of the individual symptoms (vaginal discharge, intermenstrual bleeding and vaginal blood loss related to sexual contact) was associated with T. vaginalis infection. For rectal infection (n=7) the numbers were low and no rectal symptomatology was scored.

Factors associated with Trichomonas vaginalis infection
In univariate analyses we observed

For peer review only - http://bmjopen.bmj.com/site/about/guidelines.xhtml

For peer review only

				significant associations for single status, being HIV positive, the use of hormonal contraceptives, and visiting bars (Table 1). In multivariate analysis (Table 1), having a relationship status that is single was significantly associated with a vaginal T. vaginalis infection (adjusted OR [aOR], 2.4 [95% CI, 1.5-4.0, p <0.001]) and presence of HIV infection was significantly associated (aOR, 1.6; [95% CI 1.0-2.6; p = 0.041]) T. vaginalis infection. Within women with a current vaginal T. vaginalis infection, a rectal C. trachomatis infection is significantly more present (P<0.001, OR: 4.1). The median Cp value for vaginal T. vaginalis load of infection was 28.0 (range 17.0 – 37.9). We did not observe any significant associations between the T. vaginalis load and demographic factors (Table 2). Also no association was found between the T. vaginalis load and symptoms in general. However, in the group of women with T. vaginalis infection who had vaginal discharge observed during examination, a significantly higher load was observed as compared to those without vaginal discharge (P = 0.025).
		(b) Give reasons for non-participation at each stage	-	
		(c) Consider use of a flow diagram	-	
Descriptive data	14*	(a) Give characteristics of study participants (eg demographic, clinical, social) and information on exposures and potential confounders	7	See 13
		(b) Indicate number of participants with missing data for each variable of interest	7	See 13

For peer review only - http://bmjopen.bmj.com/site/about/guidelines.xhtml

		(c) *Cohort study*—Summarise follow-up time (eg, average and total amount)		
Outcome data	15*	*Cohort study*—Report numbers of outcome events or summary measures over time		
		Case-control study—Report numbers in each exposure category, or summary measures of exposure		
		Cross-sectional study—Report numbers of outcome events or summary measures	*7*	See 13
Main results	16	(*a*) Give unadjusted estimates and, if applicable, confounder-adjusted estimates and their precision (eg, 95% confidence interval). Make clear which confounders were adjusted for and why they were included	8	Table 1
		(*b*) Report category boundaries when continuous variables were categorized		
		(*c*) If relevant, consider translating estimates of relative risk into absolute risk for a meaningful time period		

Continued on next page

For peer review only

For peer review only - http://bmjopen.bmj.com/site/about/guidelines.xhtml

Other analyses	17	Report other analyses done—eg analyses of subgroups and interactions, and sensitivity analyses	9	The median Cp value for vaginal T. vaginalis load of infection was 28.0 (range 17.0 – 37.9). We did not observe any significant associations between the T. vaginalis load and demographic factors (Table 2). Also no association was found between the T. vaginalis load and symptoms in general. However, in the group of women with T. vaginalis infection who had vaginal discharge observed during examination, a significantly higher load was observed as compared to those without vaginal discharge (P = 0.025).
**Discussion**				
Key results	18	Summarise key results with reference to study objectives	10	In this study, we describe the prevalence, risk factors, and the load of T. vaginalis infection. This is one of a few studies to report these aspects for T. vaginalis in a cohort from South Africa. We observed a vaginal T. vaginalis prevalence of 20% (95% CI 17.0-23.4) and a rectal prevalence of 1.2% (95% CI 0.6-2.4).<br>In multivariate analysis two risk factors for vaginal T. vaginalis infection were identified: having a relationship status that is single and the presence of HIV infection. In addition, within women with vaginal T. vaginalis infection, a rectal C. trachomatis infection is significantly more present. Finally, a high T. vaginalis load appears to be associated with vaginal discharge observed during examination.
Limitations	19	Discuss limitations of the study, taking into account sources of potential bias or imprecision. Discuss	12	Although this is a unique study about the epidemiology and

For peer review only - http://bmjopen.bmj.com/site/about/guidelines.xhtml

		both direction and magnitude of any potential bias		microbiological characteristics of T. vaginalis in rural South Africa with a relatively good sample size, a limitation of our study is that the number of rectal T. vaginalis infections is rather small for further analyses. As a consequence, this could have played a role in our statistical analyses. In addition, there can be a selection bias based on for instance the geographical inclusion, and self-reported symptoms can be more subjective than clinician based symptomatology. We cannot exclude potential cross-contamination between the cervico-vaginal and ano-rectal sites.
Interpretation	20	Give a cautious overall interpretation of results considering objectives, limitations, multiplicity of analyses, results from similar studies, and other relevant evidence	12	In conclusion, T. vaginalis infections are highly prevalent in the rural South African population investigated. The asymptomatic nature, potential for non-sexual transmission especially in socio-economic poor settings, and the syndromic management approach to STI control in this region are potential factors contributing to the prevalence. We observed a low rectal prevalence of which might be contamination with T. vaginalis positive vaginal fluid of the perianal surface. In multivariate analyses being single or HIV positive were identified as risk factors for vaginal T. vaginalis infection. Finally, a high T. vaginalis load seems associated with vaginal discharge observed during examination.
Generalisability	21	Discuss the generalisability (external validity) of the study results	12	See 20

For peer review only - http://bmjopen.bmj.com/site/about/guidelines.xhtml

**Other information**				
Funding	22	Give the source of funding and the role of the funders for the present study and, if applicable, for the original study on which the present article is based	12	This work was supported by the Dutch Society for Tropical Medicine (NVTG), the Netherlands. The Anova Health Institute is supported by the US President's Emergency Plan for AIDS Relief program via the US Agency for International Development under Cooperative Agreement No. AID-674-A-12-00015. The views expressed in this manuscript do not necessarily reflect those of the President's Emergency Plan for AIDS Relief or the US Agency for International Development.

*Give information separately for cases and controls in case-control studies and, if applicable, for exposed and unexposed groups in cohort and cross-sectional studies.

Note: An Explanation and Elaboration article discusses each checklist item and gives methodological background and published examples of transparent reporting. The STROBE checklist is best used in conjunction with this article (freely available on the Web sites of PLoS Medicine at http://www.plosmedicine.org/, Annals of Internal Medicine at http://www.annals.org/, and Epidemiology at http://www.epidem.com/). Information on the STROBE Initiative is available at www.strobe-statement.org.

For peer review only - http://bmjopen.bmj.com/site/about/guidelines.xhtml
